# Distinct sex-specific DNA methylation differences in Alzheimer’s disease

**DOI:** 10.1186/s13195-022-01070-z

**Published:** 2022-09-15

**Authors:** Tiago C. Silva, Wei Zhang, Juan I. Young, Lissette Gomez, Michael A. Schmidt, Achintya Varma, X. Steven Chen, Eden R. Martin, Lily Wang

**Affiliations:** 1 Division of Biostatistics, Department of Public Health Sciences, University of Miami, Miller School of Medicine, 1120 NW 14th Street, Miami, FL 33136 USA; 2 Dr. John T Macdonald Foundation Department of Human Genetics, University of Miami, Miller School of Medicine, Miami, FL 33136 USA; 3 John P. Hussman Institute for Human Genomics, University of Miami Miller School of Medicine, Miami, FL 33136 USA; 4 Sylvester Comprehensive Cancer Center, University of Miami, Miller School of Medicine, Miami, FL 33136 USA

**Keywords:** DNA methylation, Alzheimer’s disease, Sex-specific differences

## Abstract

**Background:**

Sex is increasingly recognized as a significant factor contributing to the biological and clinical heterogeneity in AD. There is also growing evidence for the prominent role of DNA methylation (DNAm) in Alzheimer’s disease (AD).

**Methods:**

We studied sex-specific DNA methylation differences in the blood samples of AD subjects compared to cognitively normal subjects, by performing sex-specific meta-analyses of two large blood-based epigenome-wide association studies (ADNI and AIBL), which included DNA methylation data for a total of 1284 whole blood samples (632 females and 652 males). Within each dataset, we used two complementary analytical strategies, a sex-stratified analysis that examined methylation to AD associations in male and female samples separately, and a methylation-by-sex interaction analysis that compared the magnitude of these associations between different sexes. After adjusting for age, estimated immune cell type proportions, batch effects, and correcting for inflation, the inverse-variance fixed-effects meta-analysis model was used to identify the most consistent DNAm differences across datasets. In addition, we also evaluated the performance of the sex-specific methylation-based risk prediction models for AD diagnosis using an independent external dataset.

**Results:**

In the sex-stratified analysis, we identified 2 CpGs, mapped to the *PRRC2A* and *RPS8* genes, significantly associated with AD in females at a 5% false discovery rate, and an additional 25 significant CpGs (21 in females, 4 in males) at *P-*value < 1×10^−5^. In methylation-by-sex interaction analysis, we identified 5 significant CpGs at *P-*value < 10^−5^. Out-of-sample validations using the AddNeuroMed dataset showed in females, the best logistic prediction model included age, estimated immune cell-type proportions, and methylation risk scores (MRS) computed from 9 of the 23 CpGs identified in AD vs. CN analysis that are also available in AddNeuroMed dataset (AUC = 0.74, 95% CI: 0.65–0.83). In males, the best logistic prediction model included only age and MRS computed from 2 of the 5 CpGs identified in methylation-by-sex interaction analysis that are also available in the AddNeuroMed dataset (AUC = 0.70, 95% CI: 0.56–0.82).

**Conclusions:**

Overall, our results show that the DNA methylation differences in AD are largely distinct between males and females. Our best-performing sex-specific methylation-based prediction model in females performed better than that for males and additionally included estimated cell-type proportions. The significant discriminatory classification of AD samples with our methylation-based prediction models demonstrates that sex-specific DNA methylation could be a predictive biomarker for AD. As sex is a strong factor underlying phenotypic variability in AD, the results of our study are particularly relevant for a better understanding of the epigenetic architecture that underlie AD and for promoting precision medicine in AD.

**Supplementary Information:**

The online version contains supplementary material available at 10.1186/s13195-022-01070-z.

## Introduction

As the US population ages, Alzheimer’s disease (AD), which occurs in approximately one out every nine people over 65 years old [1], has become one of the most prominent and most costly public health problems for older adults [2]. AD is characterized by great heterogeneity in clinical presentation, progression, and neuropathology [3]. Sex has increasingly been recognized as an important factor contributing to the phenotypic heterogeneity in AD [4–8]. Almost two thirds of AD patients in the USA are women [9]. After diagnosis, women also progress faster with more rapid cognitive and functional decline [10, 11]. On the other hand, it has also been reported men with AD have an increased risk of death [12–14].

AD is a complex disease, with the disease likely influenced by a complicated interplay of genetic and environmental factors such as smoking [15], diet [16], and exercise [17]. DNA methylation (DNAm) is an epigenetic mechanism that regulates gene expression without altering DNA sequence, and it is susceptible to environmental factors that modify the risk of diseases [18]. Alterations of DNA methylation levels have been shown to be involved in many diseases, including AD [19–24]. Encouragingly, from a biomarker perspective, dysregulated DNAm has been observed as an early feature of AD neuropathology in the brain [19] and can also be detected in the blood of AD subjects [25–31].

Several previous studies have examined differential methylation between the sexes. McCarthy et al. meta-analyzed 76 DNAm studies across different tissues and identified 184 autosomal CpGs significantly associated with sex, which were enriched in RNA splicing and DNA repair [32]. More recently, Xu et al. and Xia et al. identified genes with differential methylation between males and females in the brain prefrontal cortex and found that many of these genes participate in protein synthesis [33] and overlap with genes and pathways involved in psychiatric disorders (autism, depression, and schizophrenia) [34]. To understand the sex-specific regulatory mechanisms in aging, the strongest risk factor for AD, McCartney et al. analyzed large cohorts of the Generation Scotland study and identified DNAm loci in the blood having sex-specific associations with age, many of which were located on the X-chromosome [35]. With regard to AD, we recently meta-analyzed more than 1000 prefrontal cortex brain samples and identified a number of sex-specific AD neuropathology-associated DNAm differences, which were enriched in divergent biological processes such as integrin activation in females and complement activation in males [36].

However, currently, sex-specific DNAm differences in the blood samples of AD subjects, which can be accessed relatively easily in living individuals, are still not well characterized, and the feasibility of their use as biomarkers for AD remains to be determined. To fill this knowledge gap, we performed sex-specific meta-analyses of DNAm data from two large AD epigenome-wide association studies (EWAS) measured in blood samples to identify DNAm differences associated with AD in a sex-specific manner, and then evaluated methylation-based risk prediction models as potential biomarkers for diagnosing AD using an independent dataset. Therefore, this study falls in the realm of description and prediction [37]. Within each dataset, to identify sex-specific differences in AD, we employed two complementary approaches, a sex-stratified analysis that examined methylation to AD associations in female and male samples separately, and a methylation-by-sex interaction analysis that compared the magnitude of these associations between different sexes. To identify sex-specific DNAm differences associated with both AD neuropathology in the brain as well as AD diagnosis in the blood, we also integrated the blood sample datasets with four additional cohorts of brain samples in a cross-tissue meta-analysis. Moreover, we performed integrative analyses with gene expression data and GWAS data to prioritize the DNA methylation differences with functional significance. Finally, we developed sex-specific methylation-based risk prediction models for AD and evaluated their feasibility for diagnosing AD in an external blood sample dataset. As sex is a strong factor in influencing inter-personal variabilities in AD, the results of this study provide a valuable resource for promoting precision medicine in AD.

## Methods

### Study cohorts

For sex-specific meta-analysis of blood samples, we analyzed data from a total of 1284 whole blood samples (632 females and 652 males) from the ADNI [38] (adni.loni.usc.edu) and AIBL [24] (GEO accession: GSE153712) studies. The external validation samples included 171 whole blood samples (107 females and 64 males) from the AddNeuroMed study [28] (GEO accession: GSE144858). To avoid including early-onset AD patients, we included only samples from subjects older than 65 years.

### Preprocessing of DNA methylation data

All DNAm samples from AIBL and ADNI were measured by the same Illumina HumanMethylation EPIC beadchip, which included more than 850,000 CpGs, and all DNAm samples from AddNeuroMed were measured by the Illumina HumanMethylation450 beadchip. Supplementary Table 1 shows the number of CpGs and samples removed at each quality control (QC) step. QC for CpG probes included several steps. First, we selected probes with a detection *P*-value < 0.01 for all the samples in the cohort. A small detection *P*-value corresponds to a significant difference between signals in the probes compared to background noise. Next, using function rmSNPandCH from the DMRcate R package (version 2.10.0), we removed probes that are cross-reactive [39], located close to single nucleotide polymorphism (SNPs) (i.e., a SNP with minor allele frequency (MAF) ≥ 0.01 was present in the last five base pairs of the probe). QC for samples included restricting our analysis to samples with good bisulfite conversion efficiency (i.e., ≥ 85%). In addition, principal component analysis (PCA) was used to exclude outlier samples. To this end, PCA was performed using the 50,000 most variable CpGs for each cohort, and samples within 3 standard deviations from the mean of PC1 and PC2 were selected to be included in the final sample set. For the ADNI dataset, we additionally removed samples without methylation plate information or clinical data, and randomly selected one sample among multiple technical replicates.

The quality-controlled methylation datasets were next subjected to the QN.BMIQ normalization procedure [40]. More specifically, we first applied quantile normalization as implemented in the lumi R package (version 2.48.0) to remove systematic effects between samples. Next, we applied the β-mixture quantile normalization (BMIQ) procedure as implemented in the wateRmelon R package (version 2.2.0) to normalize beta values of type 1 and type 2 design probes in the Illumina arrays.

Immune cell type proportions (B lymphocytes, natural killer cells, CD4+ T lymphocytes, monocytes, granulocytes) were estimated using the EpiDISH [41] R package (version 2.12.0). Here, as in previous analyses of blood samples [24, 38], granulocyte proportions were computed as the sum of neutrophiles and eosinophils proportions since both neutrophils and eosinophils are classified as granular leukocytes. In the AIBL dataset, because age information was not available, sample ages were estimated using the DNAm-based-age-predictor [42] (https://github.com/qzhang314/DNAm-based-age-predictor/, elastic net method). For ADNI samples, age was calculated as the difference between the date on which blood was drawn and the birthdate of the subject. We also estimated sex status using the estimateSex() function in wateRmelon R package, which agreed with the recorded sex for all the samples. For sensitivity analysis that evaluated the impact of smoking on DNAm to AD associations, we estimated smoking scores using the SSc method described in Bollepalli et al. [43] and implemented in the R package EpiSmokEr.

### Sex-specific analysis of individual datasets

To identify sex-specific DNA methylation differences in AD, we performed both a sex-stratified analysis and a methylation-by-sex interaction analysis for each blood sample dataset. In the sex-stratified analysis, we tested methylation to AD association in female and male samples separately. In methylation-by-sex interaction analysis, we analyzed both female and male samples simultaneously and compared the effects of methylation to AD associations in females and males.

More specifically, in sex-stratified analysis, for each CpG we applied the logistic regression model to female samples and male samples separately: logit (probability of AD) ~ methylation.beta + age + methylation plate + B + NK + CD4T + Mono + Gran, where the last five terms represent estimated immune cell-type proportions. In methylation-by-sex interaction analysis, we applied the logistic regression model logit (probability of AD) ~ methylation.beta + sex + sex * methylation.beta +age + methylation plate + B + NK + CD4T + Mono + Gran to samples including both sexes.

For the AIBL dataset, logistic regression models were fitted using the glm() function in R software (version 4.2.0). For the ADNI dataset, which is a longitudinal study with some subjects contributing multiple observations, we applied logistic mixed-effects models that additionally included random subject effects to account for correlations from multiple observations generated from the same subjects. Logistic mixed-effects models were fitted using Procedure GLMMIX in SAS software (version 9.4).

### Inflation assessment and correction

We estimated genomic inflation factors (lambda values) using both the conventional approach [44] and the *bacon* method [45], which is specifically proposed for a more accurate assessment of inflations in EWAS. Briefly, the bacon method uses a Bayesian algorithm to estimate a three-component normal mixture given the observed test statistics (e.g., t-statistics corresponding to the effect of methylation beta values in regression models) where one component reflects the null distribution, and two other components correspond to the positive and negative associations in the data. Mean and standard deviations of the estimated (empirical) null distribution correspond to bias and inflation of the test statistics. In males, the estimated bias are − 0.12 and − 0.006 for ADNI and AIBL datasets; in females, the estimated bias are − 0.05 and 0.04 for ADNI and AIBL datasets. For the estimated inflation, in males, the lambda values (λ) by the conventional approach were 0.65 and 1.16, and lambdas based on the bacon approach (λ.bacon) were 0.79 and 1.05 for the ADNI and AIBL cohorts, respectively. In females, the lambda values (λ) by the conventional approach were 0.51 and 1.15, and lambdas based on the bacon approach (λ.bacon) were 0.72 and 1.04 for the ADNI and AIBL cohorts, respectively.

These estimated inflation factors showed that the *P-*values of the logistic mixed effects models based on analytical formula in the analysis of ADNI dataset is overly conservative, while *P-*values of the logistic regression models in the analysis of AIBL dataset is overly liberal. Efron et al. showed that in large-scale simultaneous testing situations (e.g., when many CpGs are tested in an analysis), serious defects in the theoretical null distribution may become obvious, while empirical Bayes methods can provide much more realistic null distributions [46]. For a more accurate statistical assessment, genomic correction using the bacon method [45], as implemented in the bacon R package, was applied to obtain bacon-corrected effect sizes, standard errors, and *P-*values for each cohort. By definition, the bacon-corrected test statistics have an estimated bias of 0 and an estimated inflation factor of 1 because empirical null distributions were used in their estimation. Indeed, after bacon correction, for males, the estimated bias is − 2.47×10^−4^ and − 8.64×10^−6^, and the estimated inflation factors were λ = 1.03 and 1.05, and λ.bacon = 1.00 and 1.00, for the ADNI and AIBL datasets, respectively. For females, the estimated bias is 2.85 × 10^−3^ and 4.75 × 10^−5^, and the estimated inflation factors were λ = 0.98 and 1.05, and λ.bacon = 1.00 and 1.00 for the ADNI and AIBL cohorts, respectively.

### Meta-analysis

To meta-analyze individual CpG results across both AIBL and ADNI datasets, we used the inverse-variance weighted fixed-effects model, which was implemented in the meta R package (version 5.5.0). The estimated effect sizes and standard errors from the meta-analysis were then re-scaled to compute odds ratios for a 1% increase in beta values (i.e., increase in beta values by 0.01). We considered CpGs with a false discovery rate of less than 5% to be statistically significant. Based on our experiences and previous studies in the analysis of EWAS measured in blood [22, 38, 47], we expected our meta-analysis to be underpowered, given the modest sample sizes of the sex-specific analyses. Therefore, we also prioritized CpGs with suggestive significance at the pre-specified significance threshold of *P-*value < 1×10^−5^.

### Differentially methylated regions analysis

For region-based meta-analysis, we used the comb-p method [48]. Briefly, comb-p takes single CpG *P*-values and locations of the CpG sites to scan the genome for regions enriched with a series of adjacent low *P*-values. In our analysis, we used sex-specific meta-analysis *P*-values for the two blood sample datasets obtained above as input for comb-p. As comb-p uses the Sidak method to account for multiple comparisons, we considered DMRs with Sidak *P*-values less than 0.05 to be significant. We used parameter settings with --seed 0.05 and --dist 750 (a *P*-value of 0.05 is required to start a region and extend the region if another *P*-value was within 750 base pairs), which were shown to have optimal statistical properties in our previous comprehensive assessment of the comb-p software [49]. To help reduce false positives, we imposed two additional criteria in our final selection of DMRs: (1) the DMR also has a nominal *P-*value < 1×10^−5^; (2) all the CpGs within the DMR have consistent direction of change in estimated effect sizes, both in the meta-analyses, as well as in analysis of each individual dataset.

### Cross-tissue meta-analysis

For males, our cross-tissue meta-analysis included data from the 652 blood samples in the ADNI (*n* = 429) and AIBL (*n* = 223) datasets described above, and an additional 388 prefrontal cortex brain samples from four independent datasets, which included samples from the ROSMAP (*n* = 265), Mt. Sinai (*n* = 53), London (*n* = 43), and Gasparoni (*n* = 27) studies that we previously analyzed in our brain samples meta-analysis [21]. Similarly, for females, our cross-tissue meta-analysis included data from the 632 blood samples in the ADNI (*n* = 364) and AIBL (*n* = 268) datasets and an additional 642 prefrontal cortex brain samples from the ROSMAP (*n* = 461), Mt. Sinai (*n* = 88), London (*n* = 64), and Gasparoni (*n* = 29) studies. We used Stouffer’s Method [50], as implemented in sumz() function of R package metap, to combine weighted *z*-scores (transformed from *P-*values) in these six datasets. For each study, weights were specified based on the square root of the total number of subjects in each study [51].

### Functional annotation of significant methylation differences

Significant methylation differences at individual CpGs and DMRs were annotated using both the Illumina (UCSC) gene annotation and GREAT (Genomic Regions Enrichment of Annotations Tool) software [52] that associates genomic regions to target genes. With the default “Basal plus method,” GREAT links each gene to a regulatory region consisting of a basal domain that extends 5 kb upstream and 1 kb downstream from its transcription start site and an extension up to the basal regulatory region of the nearest upstream and downstream genes within 1 Mb. To assess the overlap between significant CpGs and DMRs (CpG location ± 250bp or DMR location) with enhancers, we used enhancer–gene maps generated from 131 human cell types and tissues described in Nasser et al. [53], available at https://www.engreitzlab.org/resources/. More specifically, we selected enhancer-gene pairs with “positive” predictions from the ABC model, which included only expressed target genes, does not include promoter elements, and has an ABC score higher than 0.015. In addition, we also required that the enhancer-gene pairs be identified in cell lines relevant to this study (https://github.com/TransBioInfoLab/AD-meta-analysis-blood/blob/main/code/annotations/Nassser%20study%20selected%20biosamples.xlsx).

### Correlations between methylation levels of significant CpGs in AD with expressions of nearby genes

To evaluate the DNA methylation effect on the gene expression of nearby genes, we analyzed matched gene expression data (measured by Affymetrix Human Genome U 219 arrays) and DNA methylation data (measured by EPIC arrays) from 145 independent male subjects and 120 independent female subjects in the ADNI study. For each sex, we considered both significant individual CpGs, CpGs located within the significant DMRs in the AD vs. CN comparison, as well as the CpGs nominated by the cross-tissue analysis.

To test the association of target gene expressions with the DNA methylation sites, we considered CpGs located in the promoter regions and distal regions separately. More specifically, for CpGs located in the promoter region (i.e., within ± 2 kb of the transcription start sites or TSS), we tested the association between CpG methylation with expression levels of the target genes. On the other hand, for CpGs in the distal regions (> 2 kb from TSS), we tested associations between CpG methylation with expression levels of ten genes upstream and downstream from the CpG. For gene expression data, when multiple probes were mapped to a gene, we used median gene expression level over all probes mapped to the gene as its gene expression level.

To reduce the effect of potential confounding, when testing methylation-gene expression associations, we first adjusted age at visit, immune cell-type proportions (for B lymphocytes, natural killer cells, CD4+ T lymphocytes, monocytes, granulocytes), and batch effects in both DNA methylation and gene expression levels separately and extracted residuals from the linear models. Immune cell-type proportions were estimated using the R/Bioconductor package EpiDISH [41] and Xcell [54] R software (https://github.com/dviraran/xCell) for DNA methylation and gene expression data, respectively. A separate robust linear model was then used to test for association between methylation residuals and gene expression residuals, adjusting for AD status.

To assess differential gene expression of the target genes in blood samples, we analyzed the ADNI gene expression dataset using a linear regression model with log (base 2) transformed gene expression level as the outcome, AD status as the main independent variable, and age, estimated cell-type proportions, and batch as covariate variables. To assess differential expression of the target genes in brain samples, we performed a fixed-effects meta-analysis of two prefrontal cortex datasets in AD [55, 56] (GEO GSE33000, *n* = 350; GEO GSE44772, *n* = 152), by combining results from differential expression analyses of the individual datasets, which adjusted age, sex and surrogate variables for cell types and were implemented using limma R package [57].

### Correlation and overlap with genetic susceptibility loci

We searched for mQTLs in the blood using the GoDMC database [58], and mQTLs in the brain using the xQTL sever [59], downloaded from http://mqtldb.godmc.org.uk/downloads and http://mostafavilab.stat.ubc.ca/xQTLServe/, respectively. To select significant blood mQTLs in GoDMC, we used the same criteria as the original study [58], that is, considering a cis *P-*value smaller than 10^−8^ and a trans *P-*value smaller than 10^−14^ as significant. The 24 LD blocks of genetic variants reaching genome-wide significance were obtained from Supplementary Table 8 of Kunkle et al. [60].

### Sex-specific methylation risk scores

We analyzed the male samples and female samples separately to identify the best-performing sex-specific methylation-based risk prediction models for AD. For each sex, the AIBL dataset (training dataset) was used to develop logistic regression models, and the prediction results were then evaluated using the AddNeuroMed dataset (testing dataset). More specifically, for each sample in the training dataset, we first computed the Methylation Risk Score (MRS) as the sum of methylation beta values for significant CpGs from the sex-specific meta-analyses described above, weighted by their estimated effect sizes in the blood sample meta-analysis. Because samples in the testing dataset (i.e., AddNeuroMed) were measured by Illumina 450k while samples in the training datasets (i.e., ADNI and AIBL) were measured by EPIC arrays, only the subset of the significant CpGs available in both training and testing datasets were included for the computation of the MRS. The logistic regression model logit (Pr (AD)) ~ MRS + age + B + NK + CD4T + Mono + Gran was fitted to the AIBL dataset using the glm() function, where the last five terms in the model are estimated immune cell proportions estimated by the EPIDISH R package [41]. Then, predict.glm() was used to apply the estimated logistic regression model to samples in the AddNeuroMed dataset. The R package pROC was used to estimate receiver operating characteristic curves (ROCs) and area under the ROC curves (AUCs) [61]. Similarly, logistic regression models with a subset of the variables (e.g., only MRS or only age) in the above model were similarly developed using the AIBL dataset and tested on the AddNeuroMed dataset. To determine if a logistic regression model predicted AD diagnosis significantly better than chance, we used the Wilcoxon rank-sum test to compare estimated probabilities for AD cases versus controls [62].

### Sensitivity analysis

In the first analysis, we evaluated the impact of smoking on methylation differences in AD. To this end, we computed smoking scores using the SSc method described in Bollepalli et al. [43], as implemented in the R package EpiSmokEr. Our expanded logistic regression model that additionally include smoking score is logit (probability of AD) ~ methylation.beta + age + methylation plate + B + NK + CD4T + Mono + Gran + smoking score. For the analysis of ADNI dataset, additional random subject effects were also included to account for multiple observations from each subject.

In the second analysis, we evaluated the impact of education on methylation differences in AD using the ADNI dataset. Our expanded logistic regression model that additionally include education is logit (probability of AD) ~ methylation.beta + age + methylation plate + B + NK + CD4T + Mono + Gran + random (subjects) + years of education. The years of education for the AD and CN subjects were also compared using Wilcoxon rank-sum test.

### Internal validation to assess the impact of years of education on methylation-based prediction model

Among the three public datasets (AIBL, AddNeuroMed, ADNI) we analyzed, information on subject education is only available in the ADNI dataset. To assess the added prediction accuracy due to education, we performed internal validations (i.e., 10-fold cross-validations) using the ADNI dataset, by comparing our best-performing models (logit (Pr (AD)) ~ MRS + age + B + NK + CD4T + Mono + Gran in females, and logit (Pr (AD)) ~ MRS + age in males) with the models that additionally include education. To obtain an independent set of samples, only the last visit of each subject in the ADNI dataset was used for this analysis. The function createFolds() in caret R package was used to divide the data into ten folds. Average AUCs over the ten iterations in the 10-fold cross-validations for the models with and without education were then estimated and compared.

## Results

### Description of study datasets

Our sex-specific meta-analysis included DNA methylation (DNAm) data measured by the Illumina EPIC arrays and generated from blood samples of 889 independent subjects (447 females and 442 males) older than 65 years of age (Table 1). The samples were collected at baseline, at 18-months follow-up in the AIBL study, and at multiple follow-up visits ranging from 6 months to 60 months in the ADNI study [38]. A total of 632 female samples (188 cases, 444 controls) and 652 male samples (239 cases, 413 controls) were included in this study. For females, the mean ages were 77 and 73 years in the ADNI and AIBL studies, respectively. Similarly, for males, the mean ages were 79 and 73 years in these two studies.

**Table 1:** Demographic information of the study datasets

Dataset	female samples			male samples		
	sample	subjects^1^	age	sample	subjects^1^	age^2^
	(N)	(N)	mean (SD)	(N)	(N)	mean (SD)
sex-specific meta-analysis						
**ADNI**						
cases	111	69	77.81 (6.57)	180	119	79.08 (6.53)
controls	253	110	76.93 (6.77)	249	100	79.12 (6.21)
Total	364	179	77.27 (6.69)	429	219	79.1 (6.37)
**AIBL**						
cases	77	77	77.1 (6.06)	59	59	76.1 (5.53)
controls	191	191	71.9 (5.07)	164	164	72.2 (4.91)
Total	268	268	73.4 (5.87)	223	223	73.3 (5.36)
validation of methylation risk scores						
**AddNeuroMed**						
cases	53	53	76.38 (5.98)	30	30	77.5 (5.3)
controls	54	54	73.26 (5.44)	34	34	74.53 (5.21)
Total	107	107	74.8 (5.9)	64	64	75.92 (5.42)

^1^ for the longitudinal ADNI dataset, sample size at last visit

^2^ for the longitudinal ADNI dataset, age was computed from samples at last visit

### Sex-stratified and methylation-by-sex interaction analyses identified complementary sex-specific DNA methylation differences in AD

**Fig. 1:**
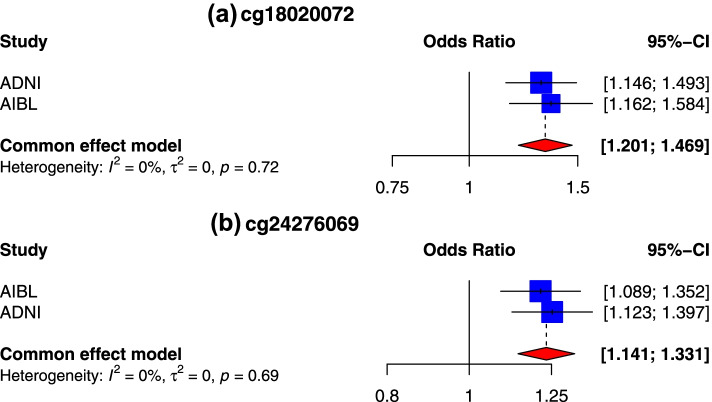
Sex-specific meta-analysis of female samples identified 2 CpGs significantly associated with AD diagnosis at 5% false discovery rate (FDR). **a** The CpG cg18020072, located on the *PRRC2A* gene, is significantly associated with AD diagnosis in females (*P*-value = 3.02 × 10^−8^, FDR = 0.023). **b** The CpG cg24276069, located on the *RPS8* gene, is also significantly associated with AD diagnosis in females (*P*-value = 9.62 × 10^−8^, FDR = 0.036). FDR: false discovery rate

**Fig. 2:**
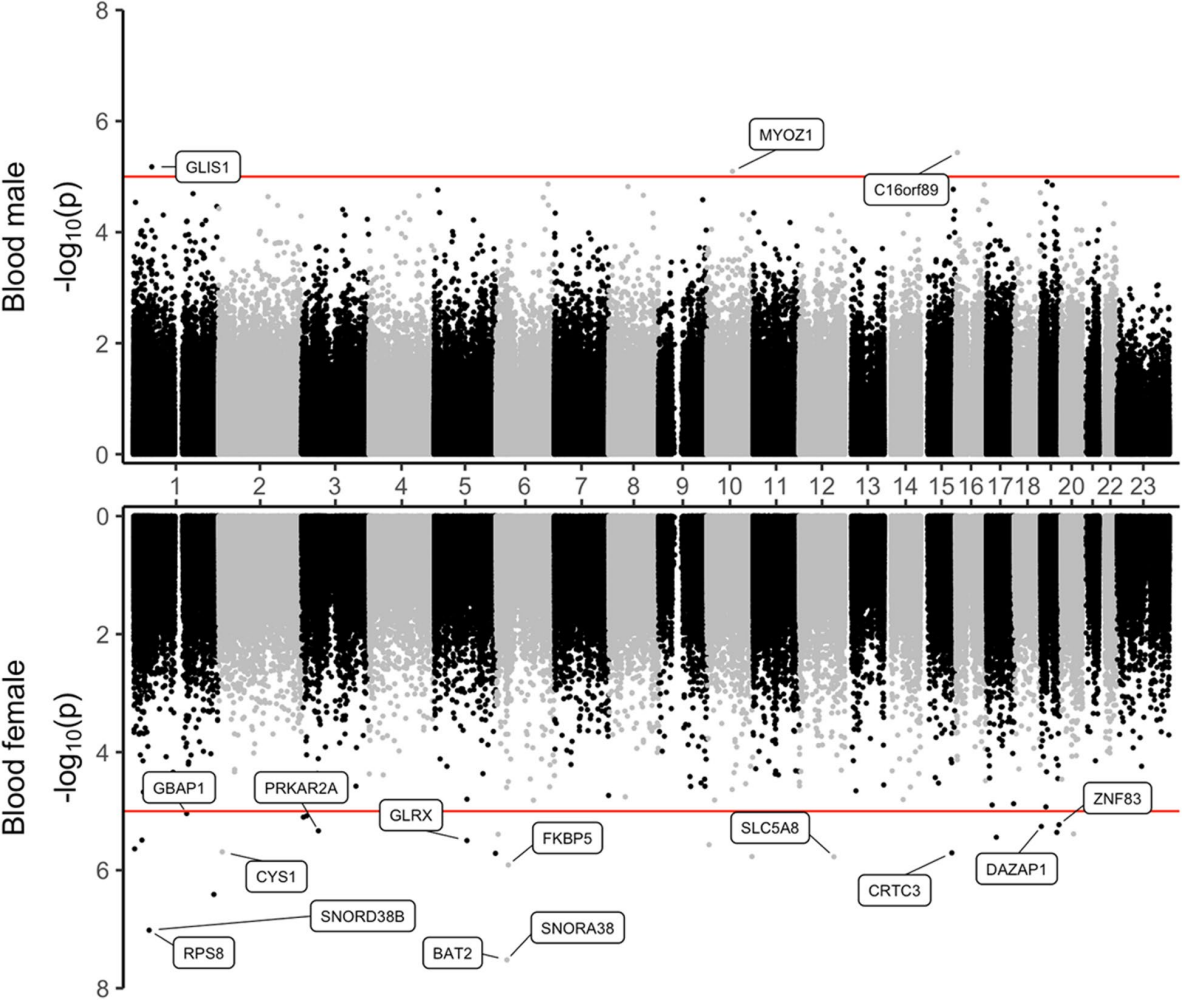
Sex-specific DNA methylation differences associated with AD diagnosis in males and females. The *X*-axis are chromosome numbers. The *Y*-axis shows -log_10_ (*P-*value) of CpGs associated with AD diagnosis in males (above *X*-axis) or in females (below *X*-axis). The genes corresponding to the CpGs that reached *P-*value < 1×10^−5^ (indicated by the red lines) are highlighted

In sex-stratified analysis, after adjusting covariate variables age, batch, and immune cell-type proportions and correcting inflation in each dataset (Methods), inverse-variance fixed-effects meta-analysis identified 2 CpGs, mapped to the *PRRC2A* and *RPS8* genes at 5% false discovery rate (FDR) (Table 2, Fig. 1) in the analysis of female samples. No CpGs reached 5% FDR in males. At the predefined suggestive threshold of *P* < 1×10^−5^, an additional 4 and 21 CpGs were identified in males and females, respectively (Fig. 2, Supplementary Table 2, Supplementary Figures 1 and 2).

**Table 2:**
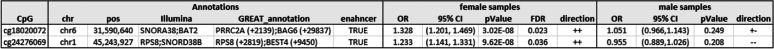
Results of sex-specific meta-analyses of the blood samples in ADNI and AIBL datasets. Inverse-variance weighted fixed-effects meta-analysis models were used to combine dataset-specific results from logistic regression models that included methylation beta values and covariate variables age, batch (i.e., methylation plate), and estimated immune cell-type proportions. In females, two CpGs were significant in the Alzheimer’s disease (AD) vs. cognitive normal groups comparison at 5% false discovery rate (FDR). No CpG reached 5% FDR in males. Annotations include the location of the CpG based on hg19/GRCh37 genomic annotation (Chr, position), nearby genes based on GREAT and Illumina gene annotations, and overlap with enhancer regions identified in Nasser et al. [53] study (enhancer). Odds ratios and their 95% confidence intervals (OR, 95% CI) describe changes in odds of AD (on the multiplicative scale) associated with a one percent increase in methylation beta values (i.e., increase in methylation beta values by 0.01) after adjusting for covariate variables. Direction indicates hypermethylation (+) or hypomethylation (−) in AD samples in the ADNI and AIBL datasets

For these 27 AD-associated CpGs, the odds ratios (ORs) for hypermethylated CpGs in AD ranged from 1.059 to 1.328 in females and 1.181 to 1.199 in males, and the ORs for hypomethylated CpGs ranged from 0.839 to 0.935 in females and was 0.677 for the only hypomethylated CpG in males (Supplementary Table 2). Overall, the majority of these CpGs were hypermethylated in AD subjects (22 CpGs), located outside CpG islands or shores (24 CpGs), or in distal regions located greater than 2k bp from the TSS (23 CpGs). Only 4 of these 27 CpGs were located in gene promoters (at *SLC5A8*, *DAZAP1*, *C16orf89* and *MYOZ1* genes). A total of 10 CpGs (all of them in females) overlapped with enhancer regions [53], which are regulatory DNA sequences that transcription factors bind to activate gene expressions [53, 63].

Using the sex-specific meta-analysis *P-*values for individual CpGs as input, the comb-p software [48] identified 41 differentially methylated regions (DMRs) in females and 24 different DMRs in males at 5% Sidak multiple comparisons corrected *P-*value (Supplementary Table 3–6). The median numbers of CpGs in these DMRs are 5 CpGs in females and 4 CpGs in males. A total of 13 DMRs (6 in females, 7 in males) overlap with enhancer regions. These DMRs are mostly distinct from the AD-associated CpGs; there is no overlap between the DMRs and significant individual CpGs in either females or males (Supplementary Fig. 3). Among the significant DMRs, about half of them (21/41 in females, 13/24 in males) are hypermethylated in AD (Table 3, Supplementary Table 3–6).

**Table 3:**
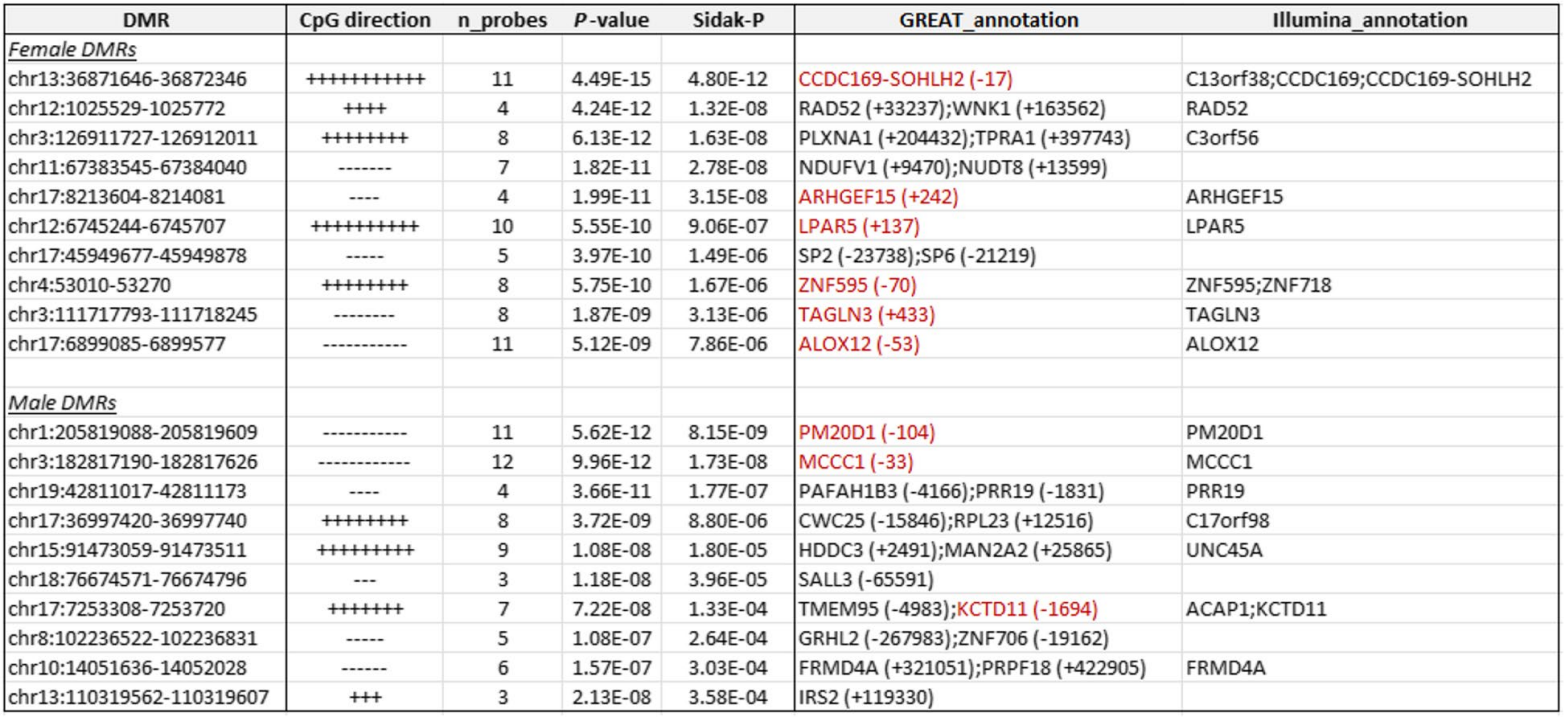
In sex-specific meta-analysis of the blood samples in ADNI and AIBL datasets, the top 10 most significant DMRs associated with Alzheimer’s disease diagnosis identified by comb-p software at 5% Sidak adjusted *P-*values. CpG direction indicates hypermethylation (+) or hypomethylation (−) in AD subjects for each CpG located within the DMR, based on effect estimate in meta-analysis. Annotations include nearby genes based on GREAT and Illumina gene annotations. Highlighted in red are promoter regions of the genes mapped by the DMRs

*Abbreviation*: *DMR* differentially methylated region

Interestingly, AD-associated DNA methylation differences are largely distinct between the sexes. There is no overlap between the significant CpGs (or DMRs) identified in females and males (Supplementary Fig. 4). Among the 27 sex-significant CpGs, there was only modest correlations between the effect estimates (i.e., odds ratios) obtained from meta-analyses of female and male samples (Spearman correlation *R* = 0.100) (Supplementary Fig. 4). About a third (9 out of 27) of the CpGs are in the same direction of change in both females and males (i.e., hypermethylated across all datasets or hypomethylated across all datasets) (Supplementary Table 2).

In methylation-by-sex interaction analysis, we identified significant interactions at 5 CpGs with *P* < 1×10^−5^ (Table 4). These CpGs mapped to the *MYO19*, *ESRRB*, *APLNR* genes, and intergenic regions. There was no overlap between significant CpGs identified in methylation-by-sex interaction and sex-stratified analyses. To understand this discrepancy, note that the interaction analysis identifies CpGs with large differences in sex-specific effect estimates that are in different directions, but these effects might not have reached the *P* < 1×10^−5^ significance threshold in sex-stratified analysis. Therefore, the results from sex-stratified analysis and methylation-by-sex interaction analysis complemented each other.

**Table 4:**
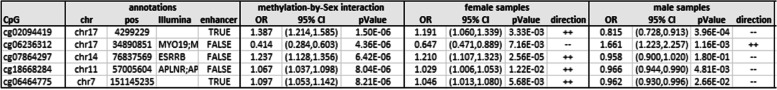
Results from meta-analysis of methylation-by-sex interaction effect in the analysis of blood samples in ADNI and AIBL datasets. Inverse-variance weighted fixed-effects meta-analysis models were used to combine dataset-specific results from logistic regression models that included methylation beta values, sex, methylation beta values*sex and covariate variables age, batch (i.e., methylation plate), and estimated immune cell-type proportions. For each CpG, annotations include the location of the CpG based on hg19/GRCh37 genomic annotation (chr, position), Illumina gene annotations, overlap with enhancer regions identified in Nasser et al. [53] study (enhancer). Odds ratios and their 95% confidence intervals (OR, 95% CI) describe changes in odds of AD (on the multiplicative scale) associated with a one percent increase in methylation beta values (i.e., increase in methylation beta values by 0.01) after adjusting for covariate variables. Direction indicates hypermethylation (+) or hypomethylation (−) in AD samples in the ADNI and AIBL datasets

### Cross-tissue meta-analysis prioritized sex-specific DNA methylation differences associated with both AD neuropathology and AD diagnosis

As changes in the brain are more relevant for cognitive disorders such as AD, we next prioritized sex-specific DNA methylation differences with changes in both blood and the brain, by performing cross-tissue analysis using two complementary approaches: (1) cross-tissue meta-analysis and (2) significant overlap.

In the first approach (i.e., cross-tissue meta-analysis), we performed a meta-analysis of the two blood sample datasets described above (i.e., AIBL and ADNI) with four additional prefrontal cortex datasets measured on brain samples, previously described by the ROSMAP [19], Mt. Sinai [23], London [20], and Gasparoni EWAS studies [64]. Supplementary Table 7 includes additional information on Braak stage, CERAD scores, clinical diagnosis, and postmortem interval for these brain samples. We previously meta-analyzed these four brain sample datasets and identified a number of CpGs and DMRs, many involved in immune processes, that are significantly associated with AD neuropathology [21, 36].

**Fig. 3:**
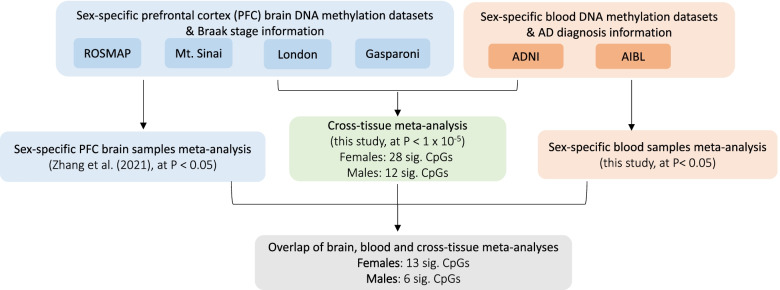
Workflow for identifying sex-specific DNA methylation differences that are associated with both AD pathology (in prefrontal cortex brain samples) and AD diagnosis (in blood samples) using cross-tissue meta-analysis approach. Results for brain sample meta-analysis were obtained from Zhang et al. [36]

In the cross-tissue meta-analysis, no CpGs reached the 5% FDR significance threshold. At *P-*value < 1 × 10^−5^, we identified 28 CpGs and 12 CpGs in females and males, respectively (Fig. 3). We then prioritized 13 CpGs in females and 6 CpGs in males by additionally requiring these CpGs to also be nominally significant (i.e., *P-*value < 0.05) in the separate sex-specific meta-analyses of brain and blood samples (Tables 5a and 6). Among them, 8 CpGs were located in enhancer regions [53]. In females, 5 CpGs are located in promoter regions of the genes *AGAP2*, *SLC44A2*, *LST1*, *VPS13D*, and *BLCAP*. In males, 2 CpGs are mapped to promoters of the *OAT* and *ADORA3* genes.

**Table 5:**
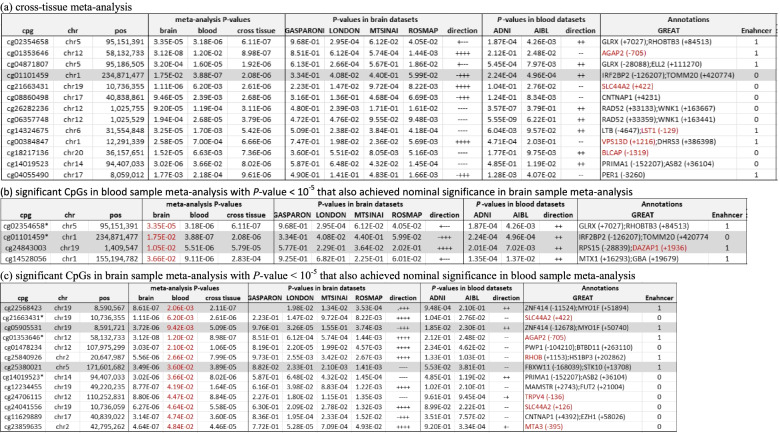
Cross-tissue analysis of female samples prioritized a total of 25 significant CpGs. (a) A total of 13 CpGs reached a *P-*value < 10^−5^ in cross-tissue meta-analyses that included both brain and blood samples, and nominal significance (i.e., *P-*value < 0.05) in sex-specific meta-analyses of each tissue. The brain sample meta-analysis results were obtained from Zhang et al. [36]; (b) A total of 4 CpGs achieved *P-*value < 10^-5^ in blood sample meta-analysis and nominal significance in brain sample meta-analysis; (c) A total of 13 CpGs achieved *P-*value < 10^-5^ in brain sample meta-analysis and nominal significance in blood sample meta-analysis. Direction indicates hypermethylation (+) or hypomethylation (-) in AD samples in individual brain or blood sample datasets. Annotations include nearby genes based on GREAT annotation and overlap with enhancer regions identified in the Nasser et al. [53] study. All but 5 significant CpG showed the same direction of change in brain and blood samples (highlighted in gray). Highlighted in red are gene promoter regions overlapped with the significant CpGs

*These CpGs were also identified in cross-tissue meta-analysis in (a)

**Table 6:**
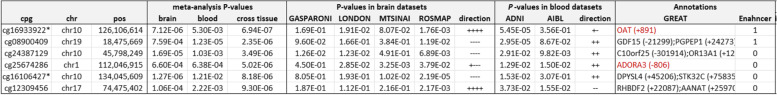
Cross-tissue analysis of male samples prioritized a total of 6 significant CpGs. These 6 CpGs reached a *P-*value < 10^−5^ in cross-tissue meta-analyses that included both brain and blood samples, and nominal significance (i.e., *P-*value < 0.05) in sex-specific meta-analyses of each tissue. The brain sample meta-analysis results were obtained from Zhang et al. [36]. Among the 6 CpGs, 2 CpGs also achieved *P-*value < 10^−5^ in brain sample meta-analysis and nominal significance in blood sample meta-analysis. Direction indicates hypermethylation (+) or hypomethylation (−) in individual brain or blood sample datasets. Annotations include nearby genes based on GREAT annotation and overlap with enhancer regions identified in the Nasser et al. [53] study. Highlighted in red are gene promoter regions overlapped with the significant CpGs

*These CpGs also achieved *P-*value < 10^−5^ in brain sample meta-analysis and nominal significance in blood sample meta-analysis

In the second approach (i.e., significant overlap), we identified CpGs that achieved *P-*value < 1×10^−5^ in the blood sample meta-analysis and nominal significance (i.e., *P-*value < 0.05) in the brain sample meta-analysis, and vice versa. In females, for the 23 significant sex-specific CpGs we discovered in blood sample meta-analysis, 4 CpGs, mapped to the promoter region of *DAZAP1* and intergenic regions, also achieved nominal significance in brain meta-analysis (Table 5b). On the other hand, for the 116 CpGs with *P-*value < 1×10^−5^ in brain meta-analysis, 13 CpGs, mapped to the promoter regions of *SLC44A2*, *AGAP2*, *RHOB*, *TRPV4*, *MTA3* genes, and intergenic regions, achieved nominal significance in blood sample meta-analysis (Table 5c). Among these 17 CpGs prioritized by the significant overlap approach, 5 CpGs were also identified by the cross-tissue meta-analysis approach.

In male samples, we did not identify any additional CpG using the significant overlap approach (Table 6). Among the 6 CpGs prioritized by cross-tissue meta-analysis, two CpGs, mapped to the *OAT* and *ADORA3* genes, also achieved *P-*value < 1×10^−5^ in brain sample meta-analysis and nominal significance in blood sample meta-analysis.

Intriguingly, among the 25 CpGs in females and 6 CpGs in males prioritized by these two complementary analyses, the majority of them (20 in females, 6 in males) showed the opposite directions of change in the brain and the blood, in which 11 CpGs in females and 2 CpGs in males were hypermethylated in the brain and hypomethylated in the blood of AD samples, and the rest were hypomethylated in the brain and hypermethylated in the blood of AD samples.

### Correlation of sex-specific DNA methylation differences in AD with expression levels of nearby genes

To better understand the functional roles of the significant DNAm differences, we examined the correlation between CpG methylation (both significant individual CpGs and CpGs within significant DMRs) and the expression levels of nearby genes. To this end, we performed integrative analysis using matched methylation and expression data measured on blood samples from 265 independent subjects (120 females and 145 males) in the ADNI study. We first removed effects in batch, age, and immune cell-type proportions in methylation and gene expression data separately. Next, for CpGs in the promoter regions (i.e., within ± 2k bp from TSS), we tested the association between the CpG with their target gene expressions. Similarly, for CpGs in the distal regions (i.e., > 2k bp from TSS), we tested the association between the CpG with ten genes upstream and ten genes downstream and within 1M bp from the CpG location.

At 5% FDR, among the significant sex-specific AD-associated CpGs and CpGs located in AD-associated DMRs, in females, DNAm at 23 CpGs (mapped to 5 DMRs) in gene promoter regions were significantly associated with the expression of their target genes, including *LGALS3BP*, *VAMP5*, *ALOX12*, *TAGLN3*, and *GABRG1* (Supplementary Table 8). Among CpGs located in distal regions, only 1 CpG (cg00271210) was significantly associated with the expression of its target gene *RNASET2* at 5% FDR.

In males, DNAm at 12 CpGs (mapped to 2 DMRs) in gene promoter regions were significantly associated with the expression of their target genes *PM20D1* and *KCTD11* (Supplementary Table 9). Among CpGs in distal regions, 13 CpGs (mapped to 5 DMRs) were significantly associated with expressions of their target genes, including *STK32C*, *TACSTD2*, *FANCA*, *OVGP1*, and *PGPEP1*.

**Fig. 4:**
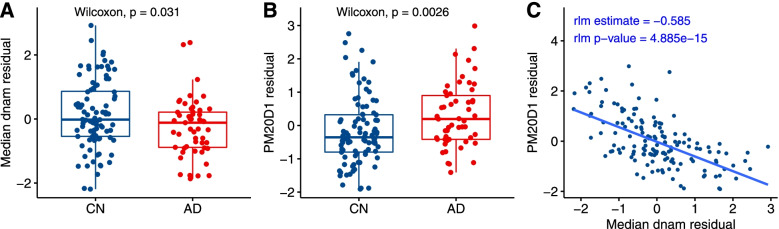
Differential DNA methylation and gene expression at the *PM20D1* gene in blood samples of male AD and cognitively normal subjects. We first removed effects of age, estimated proportions of immune cell types, and batch effects in both DNA methylation and gene expression data separately, by fitting linear regression models and extracting residuals. The results showed that **A** DNA methylation at chr1:205819088-205819609 in the promoter region of *PM20D1* is hypomethylated in AD subjects, **B** *PM20D1* gene expression levels are significantly up-regulated in AD subjects, and **C** there is a strong negative association between DNA methylation and gene expression at this locus. *Abbreviations*: dnam, DNA methylation; CN, cognitively normal; rlm, robust linear model

To further prioritize the target genes nominated by our sex-specific methylation-gene expression association analyses above, we also tested the association of the target genes with AD. In ADNI blood samples analysis, we found only 1 target gene, *PM20D1*, to be significantly upregulated in blood samples of male AD subjects (*P-*value = 2.60 ×10^−3^) (Fig. 4). In prefrontal cortex brain samples, we found several of these target genes, including *LGALS3BP*, *RNASET2*, *TAGLN3*, *VAMP5*, *ALOX12* in females, and *PGPEP1*, *KCTD11*, *STK32C*, *FANCA* in males, are differentially expressed in AD (Supplementary Table 8c, 9c). The greater number of differentially expressed genes in brain samples compared to blood samples could be due to the larger sample size of brain samples available (502 brain samples in the meta-analysis of GSE33000 and GSE44772 vs. 265 ADNI blood samples).

### Correlation and overlap with genetic susceptibility loci

To identify methylation quantitative trait loci (mQTLs) for the significant DMRs and CpGs, we next performed look-up analyses using the GoDMC database [58]. In females, among the 266 CpGs mapped to the AD-associated CpGs or located in AD-associated DMRs (Supplementary Tables 2, 3), 145 CpGs had *cis* mQTLs and 24 CpGs had both *cis* and *trans* mQTLs. In males, among the 126 CpGs mapped to the AD-associated CpGs or located in AD-associated DMRs (Supplementary Tables 2, 5), 67 CpGs had *cis* mQTLs, and 3 CpGs had both *cis* [58] and *trans* mQTLs. Among the 5 significant CpGs from interaction analysis, 2 CpGs had *cis* mQTLs. These results are consistent with the previous observation that a substantial proportion (about 45%) of the DNA methylation sites targeted by the Illumina 450k array are influenced by genetic variants in the blood [58].

Similarly, we also analyzed CpGs nominated by the cross-tissue analysis. In females, among the 25 significant CpGs prioritized in our cross-tissue analysis (Table 5), 19 CpGs had mQTLs in the blood, 7 of the 19 CpGs also had mQTLs in the brain. In the males, among the 6 significant CpGs in cross-tissue analysis (Table 6), 5 CpGs had mQTLS in the blood, and 2 of the 5 CpGs also had mQTLs in the brain. A total of 64 and 19 CpG–mQTL pairs in females and males were significant both in the analyses of brain and blood samples (Supplementary Tables 10–11).

To evaluate if these mQTLs overlapped with genetic risk loci implicated in AD, we compared them with the 24 LD blocks of genetic variants reaching genome-wide significance in a recent meta-analysis of AD GWAS [60]. We found that in females, 155 mQTLs (associated with the CpG cg14324675) overlapped with the LD block at 6:32395036-32636434, which included genetic variants mapped to the *HLA-DRB1*, *HLA-DRA*, *HLA-DRB5*, *HLA-DQA1*, and *HLA-DQB1* genes (Supplementary Table 12). In males, 864 mQTLs (associated with the CpG cg06363485) overlapped with the LD block at chromosome 6:40706366-41365821, which included genetic variants mapped to the *UNC5CL*, *TSPO2*, *APOBEC2*, *OARD1*, *NFYA*, *TREML1*, *TREM2*, *TREML2*, *TREML3P*, *TREML4*, *TREML5P*, *TREM1*, and *NCR2* genes [60] (Supplementary Table 13).

We also evaluated if the significant methylation differences overlapped with the genetic risk loci implicated in AD [60]. We found that in females, there was no overlap between AD-associated CpGs or DMRs with the genetic risk loci; in males, there was only 1 DMR that overlapped with the LD block at chromosome 6:40706366-41365821, where the *TREM2* gene is located (Supplementary Table 14). The limited commonality between genetic and epigenetic loci in AD could be due to the low power in EWAS and/or GWAS but could also reflect the relatively independent roles of genetic variants and DNA methylation in influencing AD susceptibility [65, 66].

### Out-of-sample validation of sex-specific DNA methylation differences in an independent external dataset

To evaluate the feasibility of the significant methylation differences for predicting AD diagnosis, we performed an out-of-sample validation using an independent external DNA methylation dataset measured by Illumina 450k arrays and generated by the AddNeuroMed study, which included 64 males (30 cases, 34 controls) and 107 females (53 cases and 54 controls) with ages greater than 65 years [28] (Table 1). We performed methylation risk score (MRS) analysis [67] for samples of each sex separately. More specifically, MRS was computed by summing methylation beta values of the significant sex-specific AD-associated CpGs weighted by their estimated effect sizes in the meta-analyses. Several logistic regression models were then estimated using the AIBL dataset (training dataset) and then tested on samples in the AddNeuroMed dataset (testing dataset). We considered logistic regression models with three sources of variations that might affect the prediction for AD diagnosis: age, estimated cell-type proportions for each sample, and MRS.

**Fig. 5:**
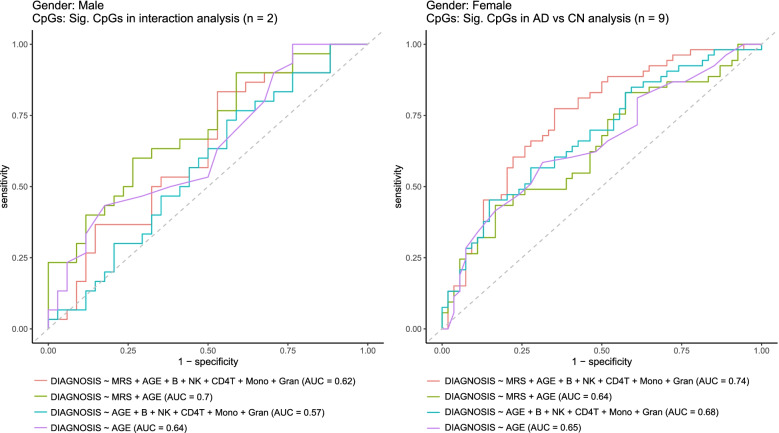
Receiver Operating Characteristic curves (ROCs) for out-of-sample validation of logistic regression models predicting AD diagnosis in males and females. The training and testing samples included sex-specific samples from AIBL and AddNeuroMed datasets, respectively. In males, the best-performing logistic regression model included age and methylation risk score (MRS) (AUC = 0.70), compared to the model with age alone (AUC = 0.64), or the model with age and estimated immune cell-type proportions (AUC = 0.57). In females, the best-performing model included age, MRS, and estimated immune cell-type proportions (AUC = 0.74), compared to the model with age and estimated immune cell-type proportions (AUC = 0.68). MRS was computed as the sum of methylation beta values for significant CpGs weighted by their estimated effect sizes from sex-specific meta-analysis of AIBL and ADNI datasets. In males, significant CpGs for the MRS included 2 CpGs with *P-*value < 10^−5^ identified in the interaction analysis that are also available in the AddNeuroMed dataset; in females, significant CpGs for MRS included 9 CpGs with *P-*value < 10^−5^ identified in AD vs. CN comparison that are also available in AddNeuroMed dataset. Abbreviations: AUC = Area Under ROC curve, AD = Alzheimer's disease, CN = cognitive normal

In females, the most predictive model include MRS, age, and estimated immune cell-type proportions (AUC = 0.74, 95% CI: 0.65–0.83), significantly more predictive than a random classifier (*P-*value = 8.42×10^−6^). In contrast, the model without MRS (i.e., only age and estimated immune cell-type proportions) has an AUC of 0.68 (Fig. 5). Because samples in the testing dataset (i.e., AddNeuroMed) are measured by Illumina 450k arrays while samples in the training datasets (i.e., ADNI and AIBL) are measured by EPIC arrays, the MRS in the best-performing model included 9 of the 23 significant CpGs with *P-*value < 10^−5^ in meta-analysis of female samples (Supplementary Table 2) that are available in both training and testing datasets.

In males, the most predictive model include MRS and age (AUC = 0.70, 95% CI: 0.56–0.82), significantly more predictive than a random classifier (*P-*value = 5.62×10^−3^). In contrast, the model without MRS (i.e., only age) has an AUC of 0.64 (Fig. 5). In the best-performing model, the MRS included 2 of the 5 significant CpGs with *P-*value < 1×10^−5^ in the meta-analysis of methylation-by-sex interaction effect (Table 4) that are available in both training and testing datasets.

Interestingly, while the best-performing prediction model for females included age, immune cell type proportions, and MRS, the best-performing prediction model for males included only age and MRS. When considered alone, immune cell type proportions achieved slightly higher prediction accuracy in females than in males (AUC_female_ = 0.59, AUC_male_ = 0.55) (Supplementary Fig. 5), which might be due to a greater change in AD-associated B cell type proportions in females (Supplementary Fig. 6). To confirm this result, we also fitted a logistic regression model to data from all three datasets (ADNI, AIBL, AddNeuroMed). This model included AD status as the outcome, main effects B cell type proportion, sex, and B cell type proportion × sex, as well as covariate variables datasets and age. The results showed a significant B cell type proportion × sex interaction (*P-*value = 0.017), indicating the associations between B-cell type proportions and AD were significantly different between males and females. While previous studies observed a decrease in B cells in the blood samples of AD patients [68–70], our findings revealed that the diminishing B cells in AD is more pronounced in females, which is also consistent with the results of another recent sex-specific analysis of gene expression data in AD [71].

We also evaluated the robustness of the best-performing sex-specific logistic regression models with additional analyses. The results indicated the prediction performance of these models in males and females remained very similar when the ADNI dataset was additionally included as a training dataset in the development of the logistic regression models, or when CpGs from AD-associated DMRs and/or significant CpGs in cross-tissue analyses are also included in the computation of MRS, where MRS weights are based on effect sizes estimated in meta-analysis of ADNI and AIBL.

### Additional sensitivity analyses

In additional to age, sex, and estimated cell-type proportions that we modeled, additional risk factors such as smoking, and education could also influence AD risk [15, 72, 73], thus may confound the methylation to AD association. To evaluate the impact of smoking on our analyses results, we repeated our meta-analysis by additionally adjusting smoking in our sex-specific logistic regression models. Because we did not have access to smoking information in the AIBL and AddNeuroMed datasets, we computed smoking scores using the SSc method, an objective measure shown to discriminate subjects with different smoking status in three independent datasets [43]. The results of our expanded logistic regression model that additionally included smoking score showed all 27 sex-specific CpGs (Supplementary Table 2) remained highly significant, with meta-analysis *P-*values ranging from 2.59 × 10^−5^ to 5.83 × 10^−8^ (Supplementary Table 15), indicating these CpGs are associated with AD independent of smoking.

Similarly, we also evaluated the impact of education by additionally including a covariate variable for years of education in the logistic regression model. Among the three public datasets (ADNI, AIBL, AddNeuroMed), we only had access to information on education in the ADNI dataset. Therefore, we compared results for the ADNI dataset using expanded model that additionally include years of education with those from our primary analysis that did not adjust for education. We found the estimated odds ratios (ORs) and *P-*values for all 27 sex-specific CpGs (Supplementary Table 2) based on the original model and expanded model to be very similar (Supplementary Table 16). Also, in the ADNI dataset, years of education did not differ significantly between CN and AD subjects in females or males (Supplementary Fig. 7), therefore is unlikely to be a confounder for AD.

## Discussion

We performed a comprehensive meta-analysis of two large independent AD blood EWAS using two complementary analyses, to identify DNA methylation differences associated with AD in a sex-specific manner. In the sex-stratified analysis, we obtained 2 CpGs, mapped to *PRRC2A* and *RPS8* genes, that reached 5% FDR in females (Table 2). An additional 21 CpGs in females and 4 CpGs in males reached *P-*value < 1×10^−5^ (Supplementary Table 2). In methylation-by-sex interaction analysis, we identified 5 CpGs, mapped to *MYO19*, *ESRRB*, *APLNR* genes, and intergenic regions with *P-*value < 1×10^−5^ (Table 4). Moreover, in region-based analysis, we also identified 41 DMRs in females and 24 DMRs in males (Supplementary Tables 3 and 5). Interestingly, there was no overlap between the significant DNA methylation differences in females and males, highlighting the distinct sex-specific epigenetic architecture underlying AD (Fig. 2, Supplementary Fig. 4).

Among genes associated with these significant DNAm differences, many were previously implicated in brain diseases. In sex-stratified analysis, the most significant CpG in females is located near the promoter region of the *PRRC2A* gene, with significant hypermethylation in AD subjects (Table 2). This result is consistent with the previous observation that deficiency in *PRRC2A* reduces the oligodendroglia population in the brain and induces hypomyelination, which leads to an impaired locomotive and cognitive functions [74–76]. The second most significant CpG is located on the *RPS8* gene, which encodes a ribosomal protein and was recently found to be significantly down-regulated in blood samples of AD patients [77]. Among genes associated with the most significant CpGs in methylation-by-sex interaction analysis, *MYO19* encodes a type of myosin associated with mitochondria [78], which are critical signaling organelles involved in the regulation of cellular metabolism and energy homeostasis. Dysregulated mitochondria dynamics have been recognized as an important contributor to AD [79, 80]. *ESRRB* encodes an estrogen-related receptor, which is involved in early development, pluripotency, and reprogramming [81]. In a recent GWAS meta-analysis, a SNP at the *ESRRB* loci was among the top 25 genetic variants most strongly associated with cognitive performance in subjects with psychotic disorders [82]. Finally, the *APLNR* gene encodes a G protein-coupled receptor (GPCR), which is a membrane protein regulating cell responses to hormones, neurotransmitters, and sensory signals. Previously, GPCRs have been implicated in the pathogenesis of Alzheimer’s disease (AD) by multiple studies [83, 84].

Also, in females, 6 out of the top 10 DMRs were mapped to promoter regions of the *CCDC169- ARHGEF15*, *LPAR5*, *ZNF595*, *TAGLN3*, and *ALOX12* genes (Table 3). Among them, *ARHGEF15* is involved in the regulation of synapse development and is significantly upregulated in AD brains [85]; *LPAR5* encodes a transmembrane receptor that is significantly downregulated during aging in human microglia, the resident immune cells of the brain [86]. In males, 3 out of the top 10 DMRs were mapped to promoter regions of the *MCCC1*, *PM20D1*, and *KCTD11* genes (Table 3). Among them, the *MCCC1* gene is involved in mitochondrial homeostasis and was shown to be associated with sporadic Parkinson’s disease in multiple GWAS [87–90]; *PM20D1* is associated with response to accumulation of amyloid-β in AD brains [91, 92]. Taken together, these results demonstrated our sex-specific meta-analyses are consistent with recent literature in brain research. In addition to implicating sex-specificity for methylation differences in genes previously known in AD (e.g., *PM20D1*), we also nominated additional differentially methylated genes that might be associated with AD (e.g., *PRRC2A* and *MCCC1*).

To better understand the relevancy of these significant CpGs, we performed several integrative analyses. Our cross-tissue meta-analysis that integrated blood DNAm samples with over 1000 additional brain samples prioritized 31 CpGs with the most significant differences in both tissues. Intriguingly, we found the majority of the CpGs (26 out of 31) had opposite directions of changes in brain and blood (Tables 5 and 6), consistent with previous studies in AD that also found DNA methylation variations in the blood, by and large, did not recapitulate those in the brain [20, 22, 93]. In addition, we also performed an integrative analysis of DNAm data with matched gene expression data in the ADNI blood samples and ROSMAP brain samples to narrow down target genes associated with the DNAm differences. We observed the strongest sex-specific association signals in a cluster of CpGs located in the promoter region of the *PM20D1* gene, which showed a strong negative association with target gene expression level (*P-*values ranging from 5.11 ×10^−15^ to 1.48×10^−10^, FDRs ranging from 2.14×10^−13^ to 5.67×10^−10^) in males (Supplementary Table 9). Moreover, the target gene *PM20D1* was also significantly upregulated in blood samples of AD subjects compared to cognitively normal subjects (Fig. 4). Previously, it was shown that in response to the neurotoxic insults in AD brains, overexpression of *PM20D1* is associated with decreased amyloid-β levels and reduces cell death both in vitro and in vivo; thus, it may have a neuroprotective role against AD [91, 92].

Moreover, we also analyzed an additional independent DNAm dataset, the AddNeuroMed dataset, to assess the predictive power of the sex-specific methylation-based prediction models, which showed higher prediction accuracy for AD diagnosis in females than males (AUC_female_ = 0.74 vs. AUC_male_ = 0.70) (Fig. 5). Our results are consistent with another recent study on sex-specific analysis of gene expression changes in AD, which found gene expression-based prediction models also performed better in females than in males [71], suggesting molecular differences in females are more predicative of AD. Another possibility for the better prediction performance could also be the larger number of female samples in the validation dataset (107 female samples vs. 64 male samples). Future validation datasets with a larger number of both female and male samples will help clarify the difference in prediction performances of the sex-specific methylation-based risk models.

To help interpret our findings, we compared our analysis results with several previous studies. The comparison with our previous sex-combined analysis of AIBL and ADNI EWAS [22] showed a small number of AD-associated CpGs and DMRs (4/23 CpGs and 1/41 DMRs in females, 1/4 CpGs, and 3/24 DMRs in males), including the DMR located in the promoter of the *PM20D1* gene we described above, were identified by both analyses (Supplementary Tables 2, 3, and 5), suggesting that DNA methylation differences at these loci were predominately driven by effects in only one sex. In contrast, the comparison with our previous sex-specific analysis of brain samples EWAS [36] showed sex-specific DNAm differences are largely distinct in the brain and the blood. Among the AD-associated CpGs and DMRs, only 1 CpG (cg02354658), located on 3’UTR of the *GLRX* gene, was significantly associated with both AD diagnosis and AD neuropathology in females. As advanced aging is the strongest factor for AD, we also compared our results with those from Yusipov et al. [94] and McCartney et al. [35], which identified 8 and 52 autosomal CpGs differentially affected by aging, but we did not find any overlap with these previous studies. A possible cause could be the small number of loci detected by these studies and ours.

This study has several limitations. First, we analyzed methylation data generated from bulk whole blood samples, which contain a complex mixture of cell types, and might have introduced substantial variability in the samples and reduced the power of our study. To reduce confounding effects due to different cell types, we included estimated cell-type proportions as covariate variables in all our analyses. Future studies that utilize single-cell technology for gene expression and DNA methylation might improve power and shed more light on the particular cell types affected by the AD-associated DNA methylation differences discovered in this study. Second, based on our previous experiences with the analysis of blood samples in AD, we pre-defined a more liberal significance threshold (i.e., *P-*value < 10^−5^) for our meta-analysis to select a small number of loci, which were then further prioritized using integrative analyses. Future studies with larger sample sizes are needed to identify DNAm differences at more stringent significance thresholds.

In the analysis of DMRs, the meta-analysis design of our study precluded many DMR analysis tools that require methylation data as input. To this end, we used comb-p software, which only required CpG locations and *P-*values as input. However, because the comb-p software may have an inflated false-positive rate [95], the resulting DMRs need to be interpreted cautiously. Third, we did not consider MCI subjects in this study because there is considerable heterogeneity among MCI subjects, with subjects converting to AD at different trajectories. As ADNI is currently conducting additional phases of the study, future analyses with a larger sample size will make it possible to detect more DNA methylation differences in AD as well as in MCI subjects. Fourth, the methylation-based prediction model could be further improved. Because DNA methylation samples in the testing dataset (AddNeuroMed) were measured by 450k arrays, which are different from the EPIC arrays used by the AIBL study, we only included the subset of significant CpGs that mapped to both types of arrays in the computation of MRS. The performance of our methylation-based prediction models can be assessed more accurately using future testing datasets measured by the EPIC arrays. Fifth, we did not include other important factors such as education attainment in our analysis, which might also influence AD [73] because we did not have access to additional covariate information in the AIBL and AddNeuroMed datasets beyond age and sex. In the ADNI dataset, we did not identify significant differences in years of education in AD subjects compared to cognitively normal controls in either females or males, which could be due to the relatively homogeneous cohort of highly educated subjects in this dataset [96] (Supplementary Fig. 7). Our internal validation using the ADNI dataset suggested additionally including years of education into our best-performing MRS-based logistic regression models did not improve prediction performance in females, and only slightly improved it in males. More specifically, a 10-fold cross-validation using the ADNI dataset showed the estimated average AUCs for the best-performing logistic regression models with and without years of education were 0.707 and 0.710 for females, and 0.650 and 0.604 for males (Supplementary Table 17). Future analysis of more diverse cohorts are needed to evaluate the impact of education on AD risk more accurately. Finally, we cannot be certain that the DNA methylation samples were obtained prior to AD diagnosis in all three publicly available datasets; therefore, the DNA methylation may represent both cause and consequence of AD, so the associations we identified do not necessarily reflect causal relationships. Additional studies are needed to establish the causality of the nominated DNA methylation markers.

## Conclusions

In summary, our meta-analysis discovered a number of novel sex-specific DNA methylation differences associated with AD in a sex-specific manner. Because of the cancelation of effects in different directions, or dilution from samples with no effect, many of the sex-specific effects were missed in our previous sex-combined analysis [22]. To assess the relevancy of our sex-specific DNAm differences, we performed several integrative analyses with additional gene expression data, DNAm data generated from brain samples, as well as assessing the feasibility of methylation-based risk score with an independent dataset. Despite the relatively modest sample size of our training dataset, the significant discriminatory classification of AD samples with our methylation-based risk prediction models demonstrated that sex-specific DNA methylation is a predictive biomarker for AD. Future studies that validate our findings in larger and more diverse community-based cohorts are needed. Overall, our study highlighted distinct sex-specific epigenetic architectures underlie AD, pointing to a pressing need for considering sex differences in the development of diagnosis and treatment strategies for AD.

## Supplementary Information

**Additional file 1: Supplementary Figure 1**. Forest plots for the 5 most significant CpGs in female samples meta-analysis of ADNI and AIBL blood sample datasets. Shown are odds ratios and confidence intervals that describe changes in odds of AD (on the multiplicative scale) associated with a one percent increase in DNA methylation beta values (i.e., increase in beta values by 0.01) after adjusting for covariate variables age at visit, batches and proportions of different blood cell types in each sample. **Supplementary Figure 2**. Forest plots for the 4 significant CpGs with *P* < 10^-5^ in male samples meta-analysis of ADNI and AIBL blood sample datasets. Shown are odds ratios and confidence intervals that describe changes in odds of AD (on the multiplicative scale) associated with a one percent increase in DNA methylation beta values (i.e., increase in beta values by 0.01) after adjusting for covariate variables age at visit, batches, and proportions of different blood cell types in each sample. **Supplementary Figure 3**. Overlap between DMRs and CpGs in males and females. There was no overlap between significant sex-specific DMRs and significant sex-specific individual CpGs in either (A) males or (B) females. **Supplementary Figure 4**. Comparison of results for DNA methylation differences in female sample meta-analysis vs. male sample meta- analysis. (A) overlap of AD-associated CpGs (B) overlap of AD-associated DMRs and (C) there was only modest correlation between effect estimates for CpG to AD associations in female meta-analysis vs. those from male meta-analysis. **Supplementary Figure 5**. Performance of different sex-specific logistic regression models for predicting AD diagnosis in out-of-sample validation. The training and testing datasets included samples from the AIBL and AddNeuroMed datasets, respectively. MRS was computed as the sum of methylation beta values for significant CpGs weighted by their estimated effect sizes from the sex-specific meta-analysis of AIBL and ADNI datasets. In males, significant CpGs for the MRS included 2 CpGs with *P* < 10^-5^ identified in the interaction analysis that are also available in the AddNeuroMed dataset; in females, significant CpGs for MRS included 9 CpGs with *P* < 10^-5^ identified in AD vs. CN comparison that are also available in AddNeuroMed dataset. Abbreviations: AUC = Area Under ROC curve, AD = Alzheimer's disease, CN = cognitive normal. **Supplementary Figure 6**. The changes in AD-associated B cell type proportions in females were more pronounced than in males in all three datasets (ADNI, AIBL, AddNeuroMed). **Supplementary Figure 7**. Comparison of years of education (PTEDUCAT) in cognitive normal (CN) and AD subjects from ADNI dataset. There was not significant association between AD status and years of education in females (*P* = 0.23) or males (*P* = 0.12), using Wilcoxon rank-sum test.

**Additional file 2: Supplementary Table 1**. Quality control (QC) information on DNA methylation samples and probes for each dataset contributing to the sex-specific meta-analyses. Under Probes QC, shown are the number of probes remaining after each QC procedure. Under Samples QC, shown are the number of samples remaining after each QC procedure. **Supplementary Table 2**. At *P* < 10^-5^, sex-specific meta-analyses identified a total of 23 CpGs and 4 CpGs signicantly associated with AD diagnosis in female samples and male samples, respectively . For each CpG, annotations include the location of the CpG based on hg19/GRCh37 genomic annotation (chr, position), nearby genes based on GREAT (GREAT_annotation), the type of associated genomic feature (RefGene_Group), Illumina gene annotations, location with respect to CpG islands (Relation_to_Island), and overlap with enhancers identified in Nasser et al. [53] study (PMID: 33828297). Inverse-variance weighted fixed-effects meta-analysis models were used to combine cohort-specific results from logistic regression models that included covariate variables age, batch, and immune cell-type proportions. A total of 9 CpGs had the same direction of change in males and females (highlighted in gray). Odds ratios (OR) describe changes in odds of AD (on the multiplicative scale) associated with a one percent increase in methylation beta values (i.e., increase in methylation beta values by 0.01) after adjusting for covariate variables. Highlighted in red are CpGs that mapped to promoter regions. 95% CI = 95% confidence interval for odds ratio. **Supplementary Table 3**. In female samples, a total of 41 DMRs were significantly associated with AD diagnosis at 5% Sidak corrected *P*-value. Among them, 6 DMRs overlapped with enhancer regions from Nasser et al. study (Nature 2021; PMID: 33828297) (Enhancer = TRUE). Highlighted in red are DMRs that mapped to promoter regions. Direction indicates hypermethylation (+) or hypomethylation (-) in AD subjects, which was determined based on hyper- or hypo- methylation of the majority of the CpGs (located within the DMR) in meta-analysis. **Supplementary Table 4**. CpGs within top 10 most significant DMRs in females. Direction indicates hypermethylation (+) or hypomethylation (-) in AD samples in the ADNI and AIBL datasets. **Supplementary Table 5**. In male samples, a total of 24 DMRs were significantly associated with AD diagnosis at 5% Sidak corrected P-value. Among them, 7 DMRs overlapped with enhancer regions from Nasser et al. study (Nature 2021; PMID: 33828297) (Enhancer = TRUE). Highlighted in red are DMRs that mapped to promoter regions. Direction indicates hypermethylation (+) or hypomethylation (-) in AD subjects, which was determined based on hyper- or hypo- methylation of the majority of the CpGs (located within the DMR) in meta-analysis. **Supplementary Table 6**. CpGs within the top 10 most significant DMRs in males. Direction indicates hypermethylation (+) or hypomethylation (-) in AD samples in the ADNI and AIBL datasets. **Supplementary Table 7**. Information on brain samples used in cross-tissue meta-analysis. **Supplementary Table 8**. Results of analysis of *female* samples. In (a) and (b), we analyzed matched DNAm-RNA from the ADNI dataset (adni.loni.usc.edu), and tested association of DNA methylation at significant CpGs with expression levels of genes located nearby. At 5% FDR, for CpGs in the promoter regions (i.e., within +/- 2k bp from TSS), DNAm at 23 CpGs (mapped to 5 DMRs) were significantly associated with expressions of their target genes. For CpGs in distal regions (>2k bp from TSS), we tested association between the CpGs with 10 genes upstream and 10 genes downstream from the CpG location. Only 1 CpG was significantly associated with expression of its target gene at 5% FDR. In (c), we performed a meta-analysis for gene expressions of the target genes using two prefrontal cortex brain samples datasets in AD (GEO accessions: GSE33000, GSE44772), to test association between gene expression and AD, adjusting for age, sex and surrogate variables for cell types. **Supplementary Table 9**. Results of analysis of *male* samples with matched DNAm-RNA data in the ADNI dataset. In (a) and (b), we tested association of DNA methylation at significant CpGs with expression levels of genes located nearby. At 5% FDR, for CpGs in the promoter regions (i.e., within +/- 2k bp from TSS), DNAm at 12 CpGs (mapped to 2 DMRs) were significantly associated with expressions of their target genes. For CpGs in distal regions (>2k bp from TSS), we tested association between the CpGs with 10 genes upstream and 10 genes downstream from the CpG location. A total of 13 distal CpGs (mapped to 5 DMRs) were significantly associated with expressions of their target genes at 5% FDR. In (c), we performed a meta-analysis for gene expressions of the target genes using two prefrontal cortex brain samples datasets in AD (GEO accessions: GSE33000, GSE44772), to test association between gene expression and AD, adjusting for age, sex and surrogate variables for cell types. **Supplementary 10**. In femlaes, a total of 64 CpG - mQTL pairs were significant in both brain and blood samples analyses. The blood mQTLs and brain mQTLs were obtained from the GoDMC database and xQTL server, respectively. **Supplementary 11**. In males, a total of 19 CpG - mQTL pairs were significant in both brain and blood samples analyses. The blood mQTLs and brain mQTLs were obtained from the GoDMC database and xQTL server, respectively. **Supplementary Table 12**. In females, a total of 155 mQTLs in the blood overlapped with the 24 GWAS nominated LD blocks in Kunkle et al. [60] (PMID: 30820047). The mQTLs in blood were obtained from the GoDMC database. Annotations for CpGs include location of the CpG based on hg19/GRCh37 genomic annotation (Chr, Position), Illumina gene annotation (UCSC_RefGene_Name), the type of associated genomic feature (UCSC_RefGene_Group), and location with respect to CpG islands (Relation_to_Island). **Supplementary Table 13**. In males, a total of 864 mQTLs in the blood overlapped with the 24 GWAS nominated LD blocks in Kunkle et al. [60] (PMID: 30820047). The mQTLs in blood were obtained from the GoDMC database. Annotations for CpGs include location of the CpG based on hg19/GRCh37 genomic annotation (Chr, Position), Illumina gene annotation (UCSC_RefGene_Name), the type of associated genomic feature (UCSC_RefGene_Group), and location with respect to CpG islands (Relation_to_Island). **Supplementary Table 14**. Overlap of AD-associated DMRs with AD GWAS loci reported in Kunkle et al. [60]. **Supplementary Table 15**. Sensitivity analysis for model that additionally adjust for smoking scores, which was computed using the SSc method as implemented in R package EpiSmokEr (PMID: 31466478). All 27 sex-specific CpGs from Supplementary Table 2 remained highly significant, with meta-analysis *P-*values ranging from 5.83 x 10^-8^ to 2.59 x 10^-5^. **Supplementary Table 16**. Sensitivity analysis comparing logistic regression model that additionally adjusts years of education vs. model not adjust education in the analysis of ADNI dataset. **Supplementary Table 17**. Results of internal validation that compared logsitic regression models with or without education effect. A 10-fold cross-validation using the ADNI dataset showed the estimated average AUCs for the best performing logistic regression models with and without education were 0.707 and 0.710 in females, and 0.650 and 0.604 in males. The MRS was computed as the sum of methylation beta values for significant CpGs weighted by their estimated effect sizes obtained in the meta-analysis. In males, significant CpGs used for the MRS included 2 out of the 5 significant CpGs in the meta-analysis of methylation-by-sex interaction effect which were also available in AddNeuroMed dataset. In females, significant CpGs used for MRS included 9 out of 23 CpGs in meta-analysis that compared AD vs. CN samples which were also available in AddNeuroMed dataset.

## Data Availability

All datasets analyzed in this study are publicly available as described in the “Methods” section. In particular, ADNI, AIBL and AddNeuroMed datasets can be accessed from http://adni.loni.usc.edu and Gene Expression Omnibus (GEO) (accession: GSE153712, GSE144858). The scripts for the analysis performed in this study can be accessed at https://github.com/TransBioInfoLab/AD-meta-analysis-blood-by-sex.
