# Supplementary material for: Distinct sex-specific DNA methylation differences in Alzheimer’s disease
Source: Alzheimers Res Ther. 2022 Sep 15;14:133. doi: 10.1186/s13195-022-01070-z (PMC9479371; doi:10.1186/s13195-022-01070-z)
Supplement: Supplementary file 1 — Additional file 1: Supplementary Figure 1. Forest plots for the 5 most significant CpGs in female samples meta-analysis of ADNI and AIBL blood sample datasets. Shown are odds ratios and confidence intervals that describe changes in odds of AD (on the multiplicative scale) associated with a one percent increase in DNA methylation beta values (i.e., increase in beta values by 0.01) after adjusting for covariate variables age at visit, batches and proportions of different blood cell types in each sample. Supplementary Figure 2. Forest plots for the 4 significant CpGs with P < 10-5 in male samples meta-analysis of ADNI and AIBL blood sample datasets. Shown are odds ratios and confidence intervals that describe changes in odds of AD (on the multiplicative scale) associated with a one percent increase in DNA methylation beta values (i.e., increase in beta values by 0.01) after adjusting for covariate variables age at visit, batches, and proportions of different blood cell types in each sample. Supplementary Figure 3. Overlap between DMRs and CpGs in males and females. There was no overlap between significant sex-specific DMRs and significant sex-specific individual CpGs in either (A) males or (B) females. Supplementary Figure 4. Comparison of results for DNA methylation differences in female sample meta-analysis vs. male sample meta- analysis. (A) overlap of AD-associated CpGs (B) overlap of AD-associated DMRs and (C) there was only modest correlation between effect estimates for CpG to AD associations in female meta-analysis vs. those from male meta-analysis. Supplementary Figure 5. Performance of different sex-specific logistic regression models for predicting AD diagnosis in out-of-sample validation. The training and testing datasets included samples from the AIBL and AddNeuroMed datasets, respectively. MRS was computed as the sum of methylation beta values for significant CpGs weighted by their estimated effect sizes from the sex-specific meta-analysis of AIBL and ADN [file 13195_2022_1070_MOESM1_ESM.pdf]

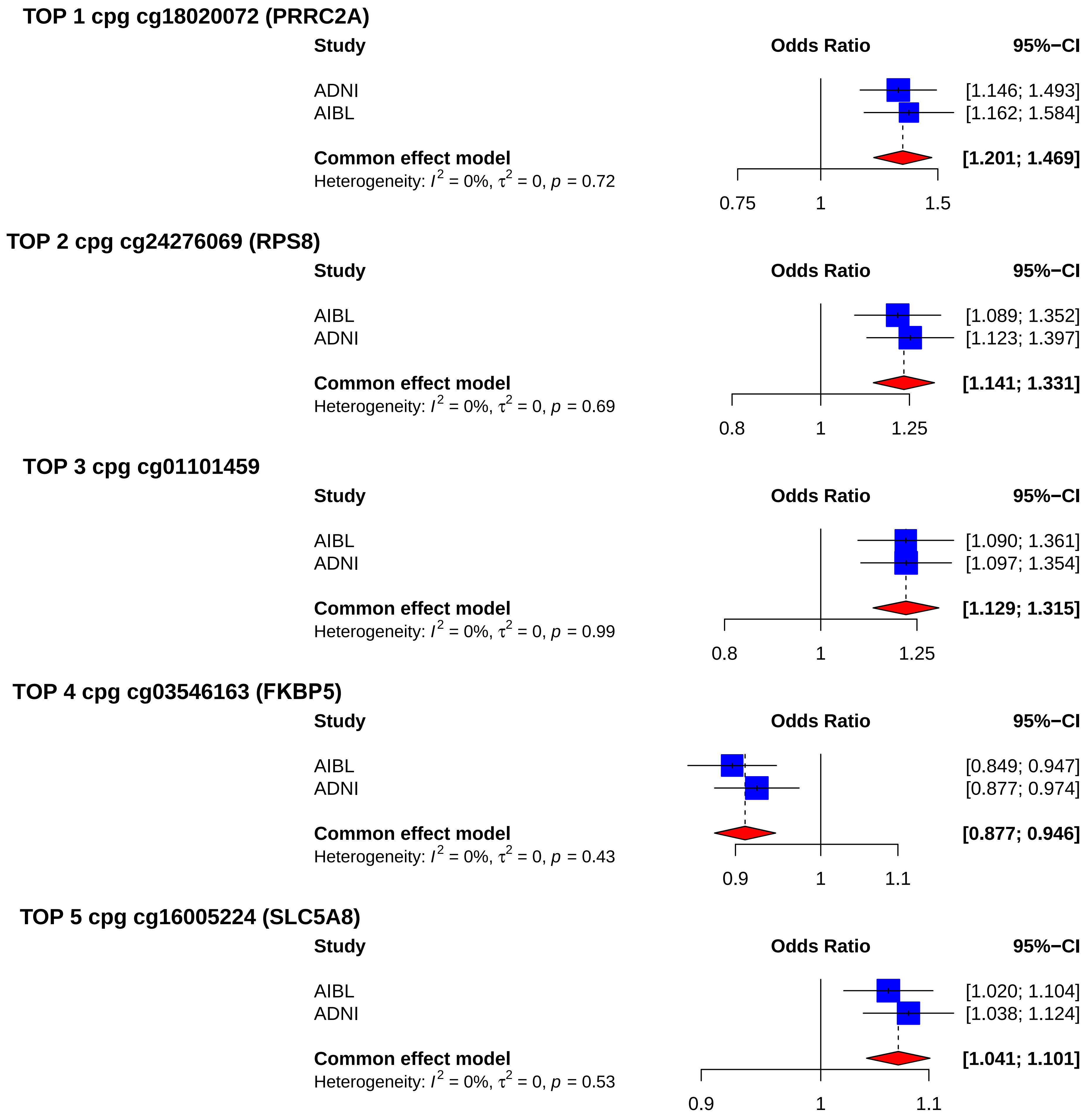

Supplementary Figure 1 Forest plots for the 5 most significant CpGs in female samples meta-analysis of ADNI and AIBL blood sample datasets. Shown are odds ratios and confidence intervals that describe changes in odds of AD (on the multiplicative scale) associated with a one percent increase in DNA methylation beta values (i.e., increase in beta values by 0.01) after adjusting for covariate variables age at visit, batches and proportions of different blood cell types in each sample.

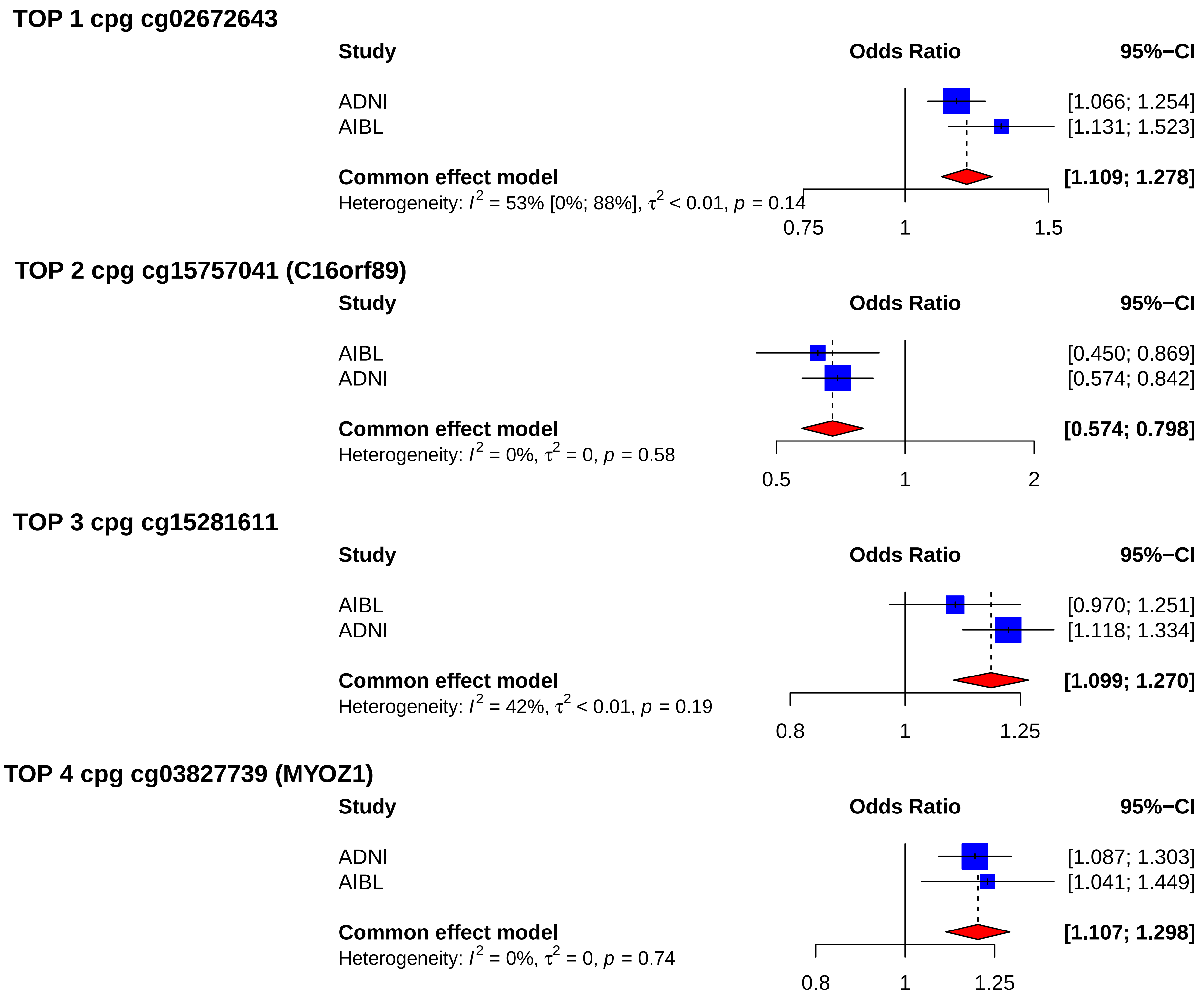

Supplementary Figure 2 Forest plots for the 4 significant CpGs with $P < 10^{-5}$ in male samples meta-analysis of ADNI and AIBL blood sample datasets. Shown are odds ratios and confidence intervals that describe changes in odds of AD (on the multiplicative scale) associated with a one percent increase in DNA methylation beta values (i.e., increase in beta values by 0.01) after adjusting for covariate variables age at visit, batches, and proportions of different blood cell types in each sample.

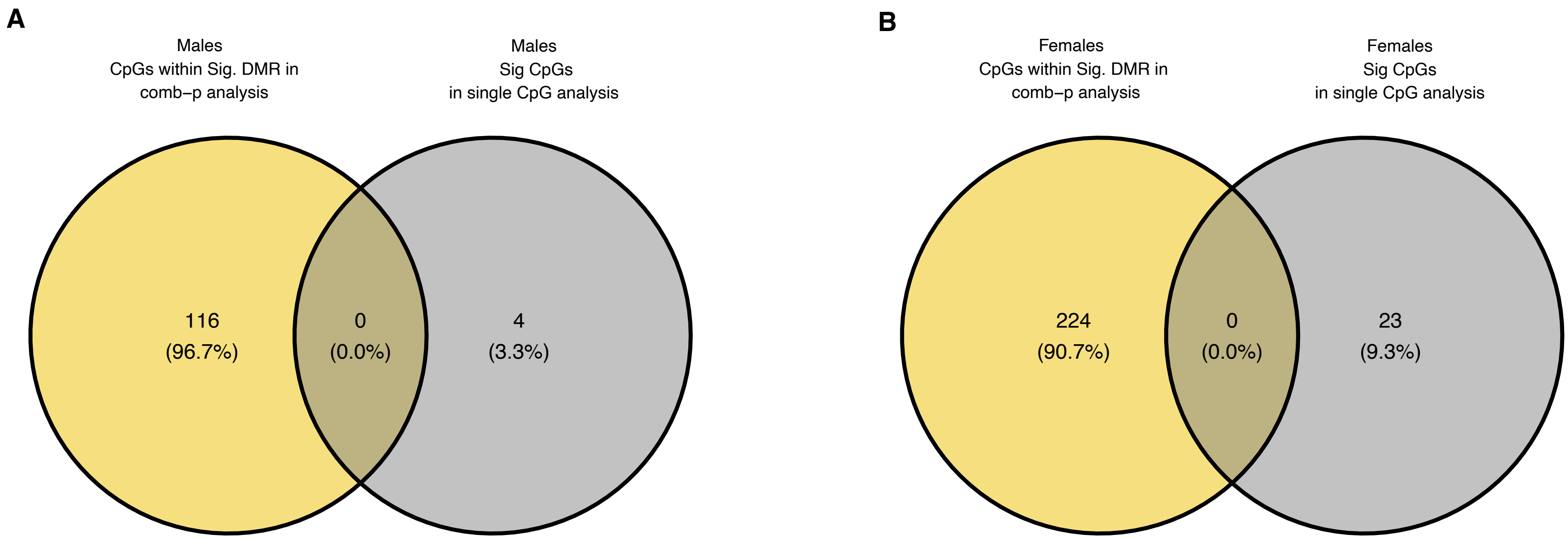

Supplementary Figure 3 Overlap between DMRs and CpGs in males and females. There was no overlap between significant sex-specific DMRs and significant sex-specific individual CpGs in either **(A)** males or **(B)** females.

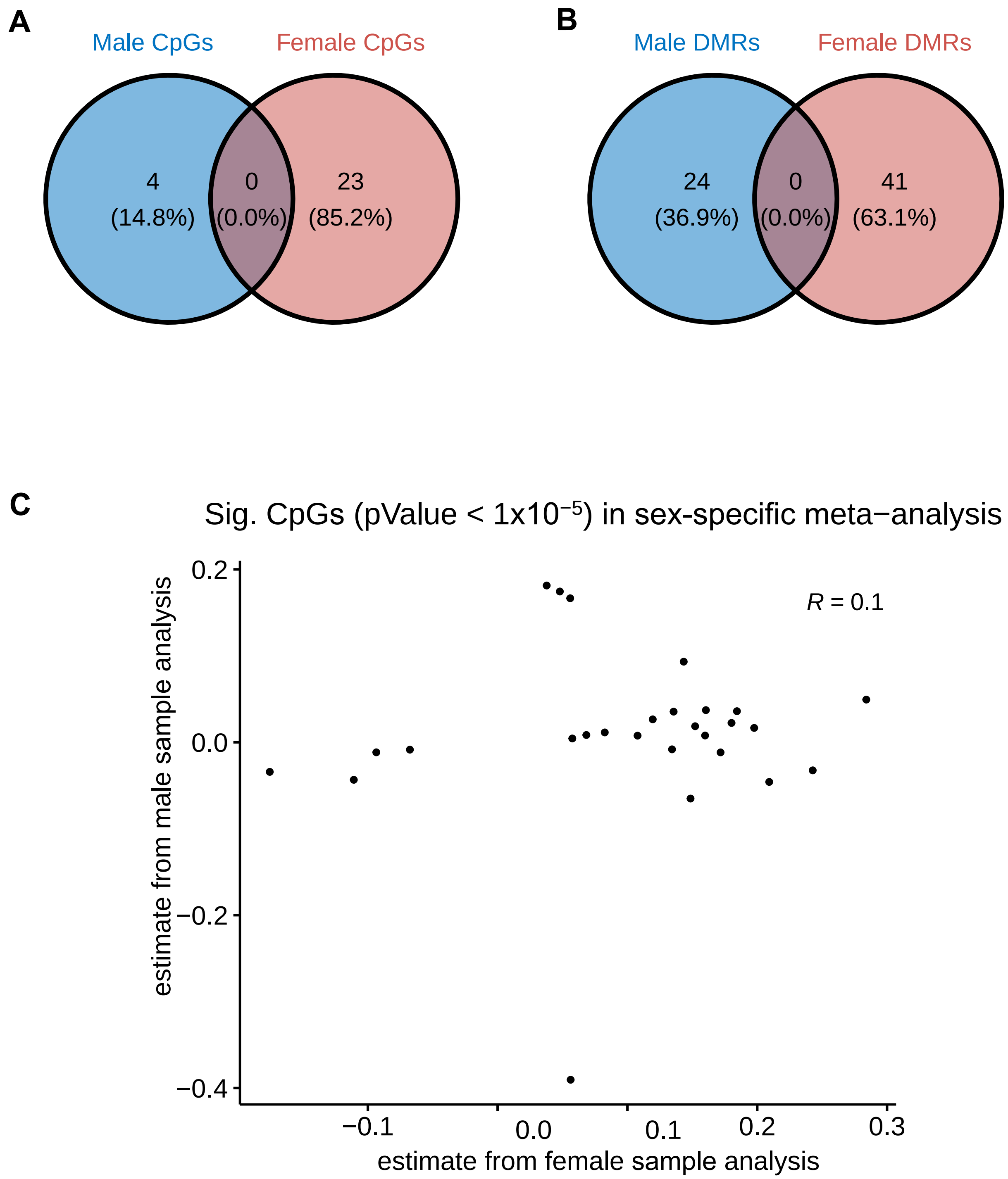

Supplementary Figure 4 Comparison of results for DNA methylation differences in female sample meta-analysis vs. male sample meta- analysis. **(A)** overlap of AD-associated CpGs **(B)** overlap of AD-associated DMRs and **(C)** there was only modest correlation between effect estimates for CpG to AD associations in female meta-analysis vs. those from male meta-analysis

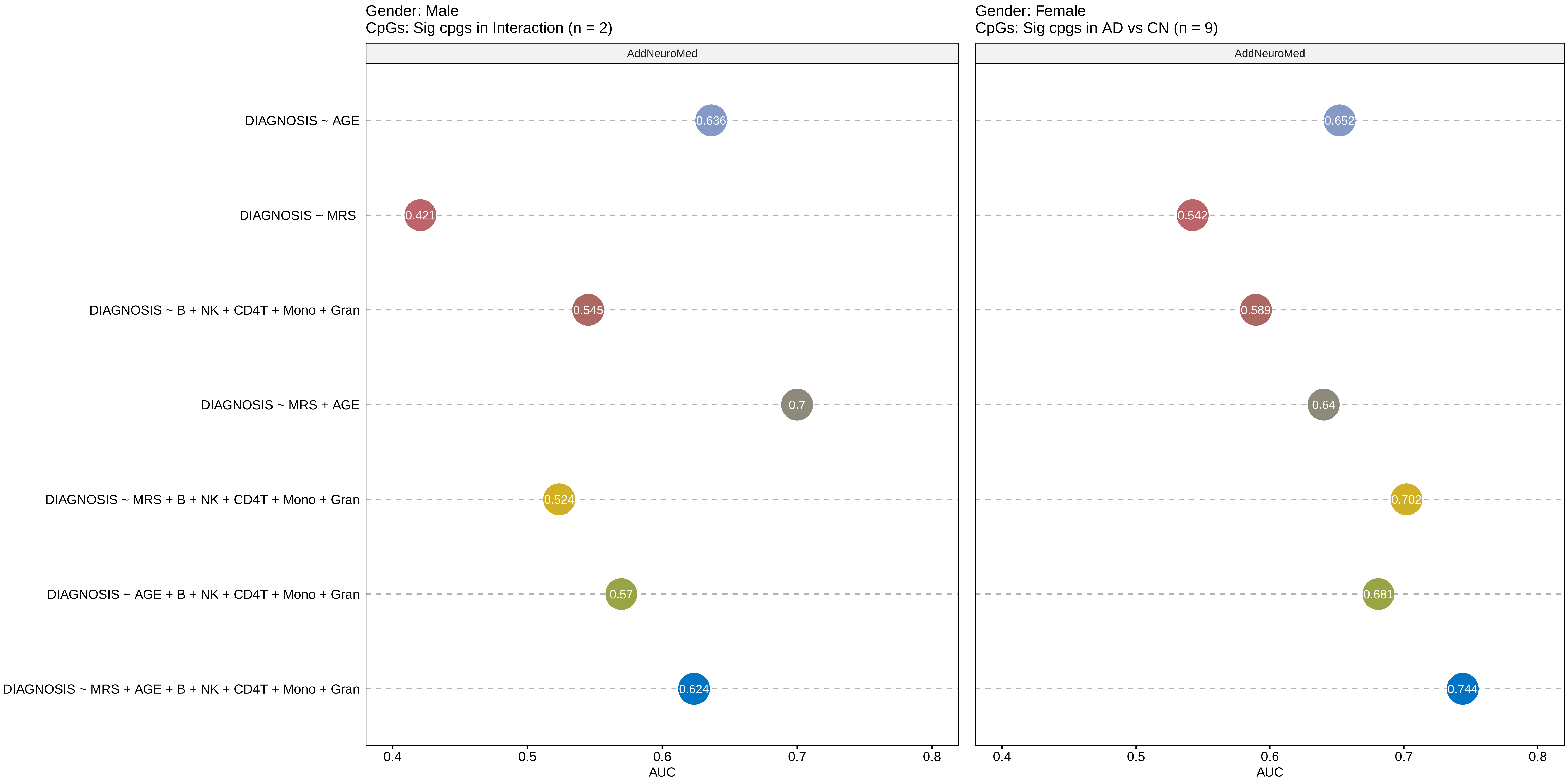

Supplementary Figure 5 Performance of different sex-specific logistic regression models for predicting AD diagnosis in out-of-sample validation. The training and testing datasets included samples from the AIBL and AddNeuroMed datasets, respectively. MRS was computed as the sum of methylation beta values for significant CpGs weighted by their estimated effect sizes from the sex-specific meta-analysis of AIBL and ADNI datasets. In males, significant CpGs for the MRS included 2 CpGs with $P < 10^{-5}$ identified in the interaction analysis that are also available in the AddNeuroMed dataset; in females, significant CpGs for MRS included 9 CpGs with $P < 10^{-5}$ identified in AD vs. CN comparison that are also available in AddNeuroMed dataset. **Abbreviations: AUC** = Area Under ROC curve, **AD** = Alzheimer's disease, **CN** = cognitive normal

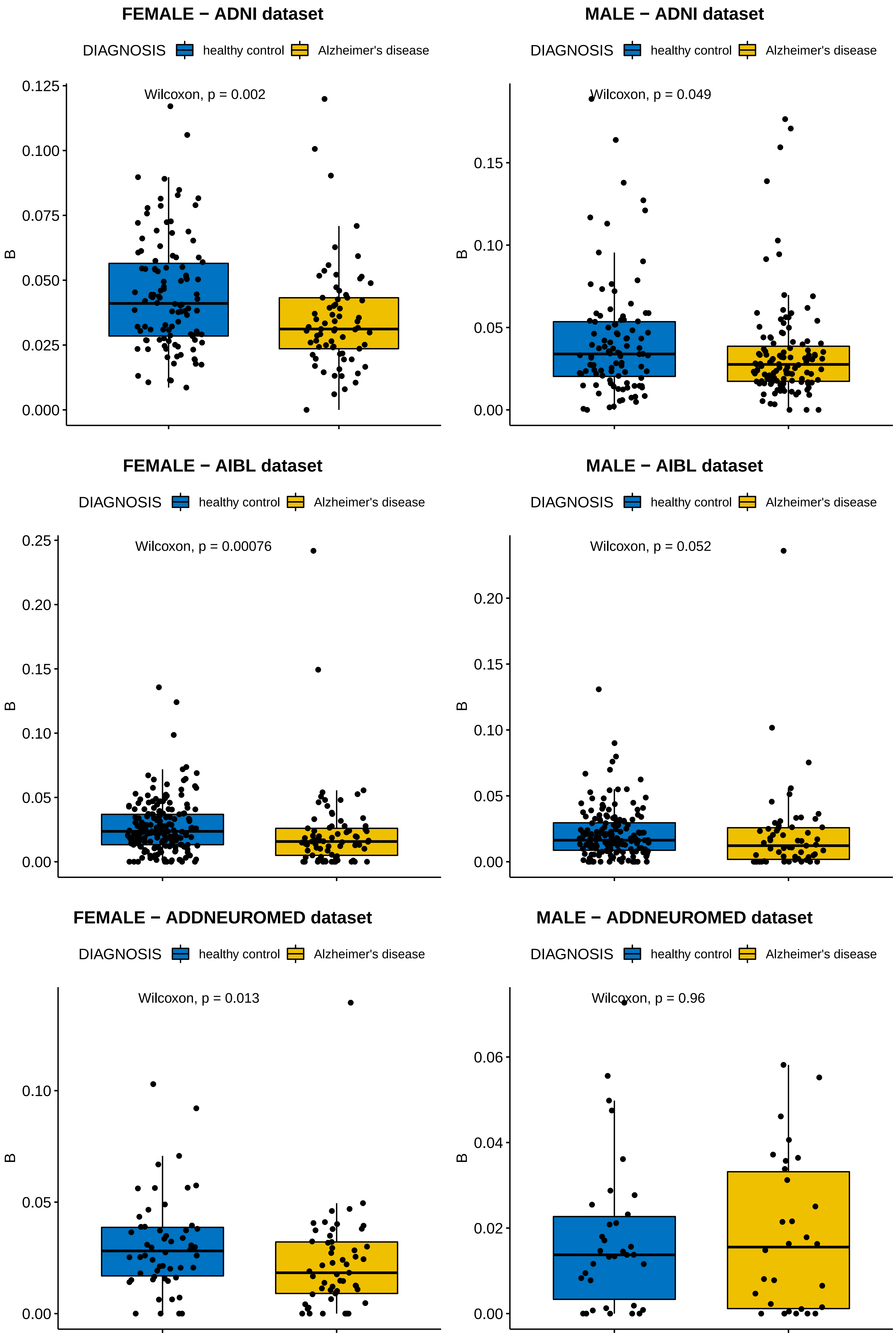

Supplementary Figure 6 The changes in AD-associated B cell type proportions in females were more pronounced than in males in all three datasets (ADNI, AIBL, AddNeuroMed).

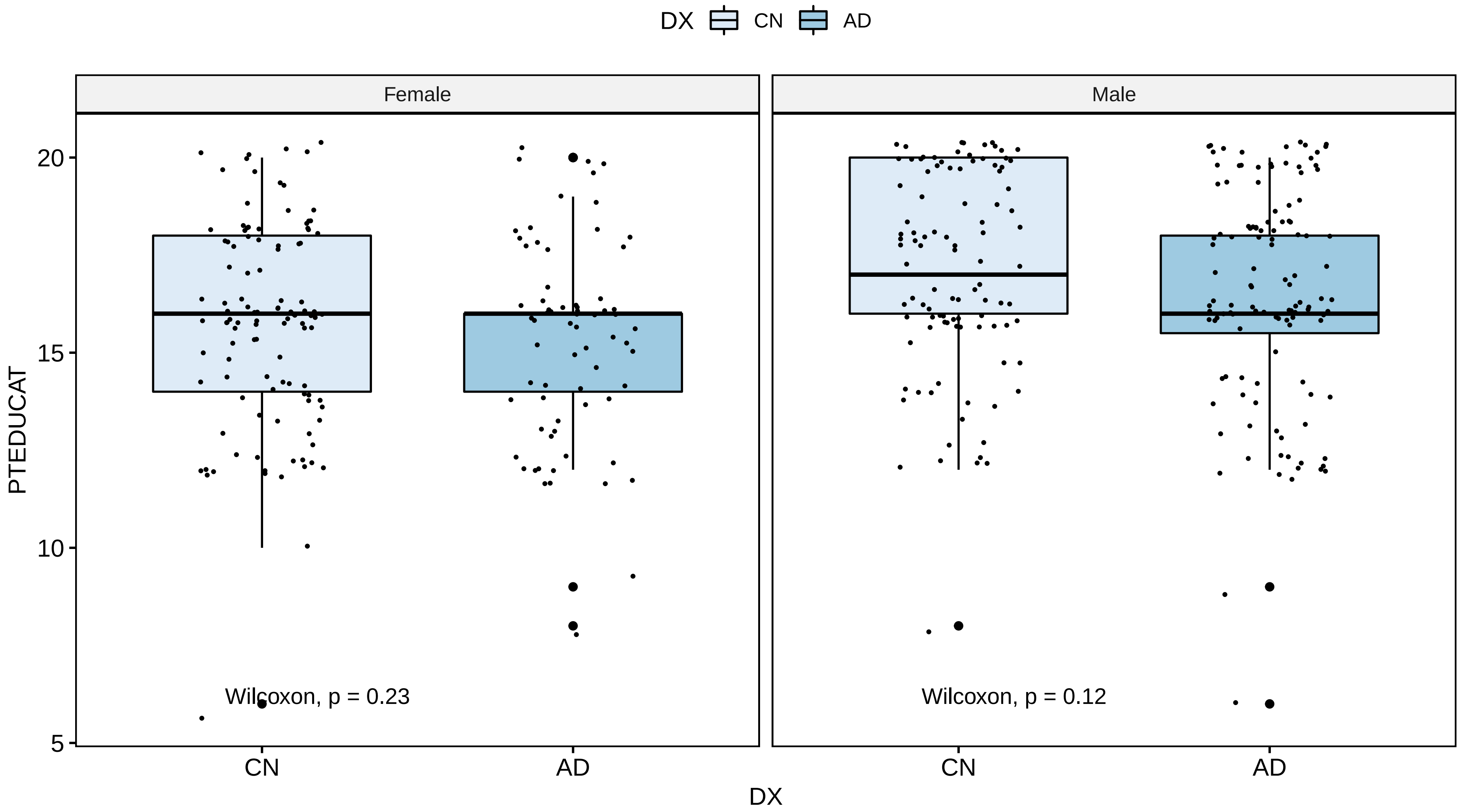

Supplementary Figure 7 Comparison of years of education (PTEDUCAT) in cognitive normal (CN) and AD subjects from ADNI dataset. There was not significant association between AD status and years of education in females (*P* = 0.23) or males (*P* = 0.12), using Wilcoxon rank-sum test.
